# Architecture of TFIIIC and its role in RNA polymerase III pre-initiation complex assembly

**DOI:** 10.1038/ncomms8387

**Published:** 2015-06-10

**Authors:** Gary Male, Alexander von Appen, Sebastian Glatt, Nicholas M. I. Taylor, Michele Cristovao, Helga Groetsch, Martin Beck, Christoph W. Müller

**Affiliations:** 1 European Molecular Biology Laboratory (EMBL), Structural and Computational Biology Unit, Meyerhofstrasse 1, Heidelberg 69117, Germany

## Abstract

In eukaryotes, RNA Polymerase III (Pol III) is specifically responsible for transcribing genes encoding tRNAs and other short non-coding RNAs. The recruitment of Pol III to tRNA-encoding genes requires the transcription factors (TF) IIIB and IIIC. TFIIIC has been described as a conserved, multi-subunit protein complex composed of two subcomplexes, called τA and τB. How these two subcomplexes are linked and how their interaction affects the formation of the Pol III pre-initiation complex (PIC) is poorly understood. Here we use chemical crosslinking mass spectrometry and determine the molecular architecture of TFIIIC. We further report the crystal structure of the essential TPR array from τA subunit τ131 and characterize its interaction with a central region of τB subunit τ138. The identified τ131–τ138 interacting region is essential *in vivo* and overlaps with TFIIIB-binding sites, revealing a crucial interaction platform for the regulation of tRNA transcription initiation.

## Abstract (web summary)

TFIIIC is a RNA polymerase III-specific general transcription factor complex essential for tRNA synthesis. Here the authors combine chemical crosslinking/mass spectrometry and X-ray crystallography to define the architecture of TFIIIC and suggest a model for the assembly of pre-initiation complexes at tRNA genes.

## Graphical abstract

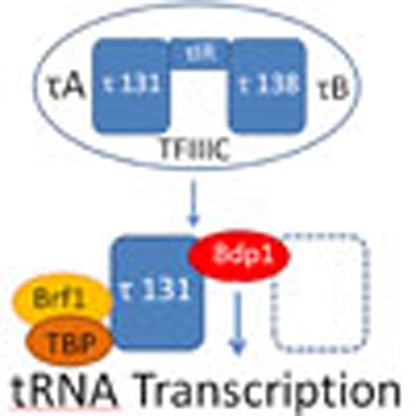

Eukaryotes require the multi-subunit Pol III to transcribe genes encoding small, non-coding RNAs including tRNAs^1^. Recruitment of Pol III to these genes requires the essential transcription factors TFIIIB and TFIIIC^2^. TFIIIB is assembled from three subunits, namely Brf1, Bdp1 and TBP^3^. TFIIIC harbours six proteins in yeast, and has a molecular weight of approximately 500 kDa (ref. 4). The six subunits of yeast TFIIIC are partitioned into two DNA-binding subcomplexes (τA and τB). Each subcomplex binds to its respective, highly conserved promoter sequence, named ‘A box' and ‘B box'^5^,^6^. The binding of these intragenic promoters by TFIIIC permits the assembly of TFIIIB, upstream of the transcription start site, which subsequently leads to the recruitment of Pol III, the formation of a PIC and finally to transcription.

Both *in vitro* and *in vivo* studies have demonstrated that TFIIIC is a highly flexible protein complex that can accommodate the varying lengths of DNA sequence between the A and B boxes of different tRNA genes^7^,^8^. It is hypothesized that a flexible linker between the τA and τB subcomplexes allows TFIIIC to fulfil these requirements^9^,^10^. In yeast, τA is composed of τ131 (Tfc4), τ95 (Tfc1) and τ55 (Tfc7) and τB of τ138 (Tfc3), τ91 (Tfc6) and τ60 (Tfc8)^5^. All six subunits are essential *in vivo*^3^. In human, orthologues of all six yeast subunits have been identified, with sequence similarity highest amongst the τA subunits^11^.

While a significant amount of high-resolution structural and functional information exists for some individual TFIIIC subunits^12^,^13^,^14^, a detailed understanding of how the τA and τB subcomplexes are connected and which subunits could act as a flexible linker remains elusive (Fig. 1a). τ95 of τA has been proposed as a linker, based on co-immunoprecipitation experiments that show binding of this subunit to the τB subunits τ138 and τ91 (ref. 15). A genetic study suggests that the two largest TFIIIC subunits, τ138 and τ131, are responsible for connecting τA and τB^16^. The τ138 subunit is currently the least well-characterized subunit of TFIIIC. Photochemical crosslinking studies support the view that τ138 recognizes the B box promoter of tRNA genes^17^, as does the mutation of a highly conserved glycine residue (G349E) within τ138 that dramatically reduces the affinity of TFIIIC for tDNA^18^. Interestingly, this mutation can be suppressed by point mutations within the τ131 subunit, suggesting a direct physical interplay between these two subunits^16^. τ131 shows the highest sequence conservation of the TFIIIC subunits and is predicted to contain several tetra-trico peptide repeats (TPRs)^19^. TPRs typically contain 34 amino acids arranged into two antiparallel alpha-helices, with proteins often containing several of these motifs in an array^20^. This arrangement can lead to an extended, right-handed super-helical structure, which can provide extended binding surfaces for other subunits within a protein assembly^21^. τ131 fulfills this role within TFIIIC, binding to subunits Brf1 and Bdp1 in a stepwise mechanism to help assemble TFIIIB at tRNA genes, utilizing overlapping sites on an amino-terminal (N-terminal) ‘TPR array'^19^,^22^,^23^,^24^,^25^,^26^,^27^,^28^. It has been further hypothesized that the relative positioning of the TPRs and the extended τ131 N terminus mask binding sites within the TPR array, leading to an auto-inhibited τ131 state that must be relieved to allow Brf1 and Bdp1 binding^27^,^28^. These rate-limiting steps in Pol III PIC formation may be overcome, at least in part, by flexibility within τ131. Indeed, previous studies suggest conformational changes take place within τ131 on the binding of Brf1 and Bdp1 (refs 25, 29). Finally, τ131 has also been shown to bind Pol III subunits Rpc53 and ABC10α^30^,^31^. Molecular details of the interactions of τ131 with τA or τB subunits have not been reported, although in humans it has been shown that the τ131 orthologue (TFIIIC102) interacts with the τ95 orthologue (TFIIIC63), again requiring the conserved TPR array^32^.

We set out to characterize the molecular architecture of TFIIIC and identify the τA–τB linker region within TFIIIC using a combination of structural and biochemical approaches. Our findings implicate a central role for the TPR array of τ131 in linking τA, τB and TFIIIB to regulate the formation of the Pol III PIC.

## Results

### The τ131 TPR array crosslinks to a central region of τ138

**Figure 1:**
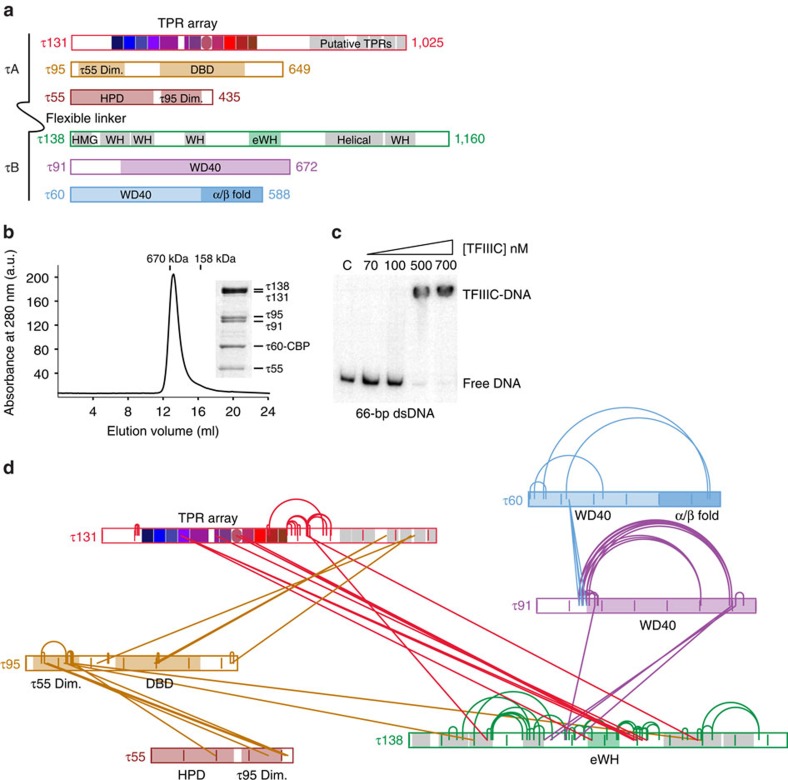
XL-MS of purified, endogenous TFIIIC reveals a link between τA and τB. (**a**) Schematic representation of the six subunits of *S. cerevisiae* TFIIIC. The amino-acid lengths of the subunits are labelled at the C terminus. Domains of which crystal structures are available are highlighted. τ55 and τ95 Dim.=τ55 and τ95 dimerization domains; DBD, DNA-binding domain; HPD, histidine phosphatase domain. The tetra-trico peptide (TPR) array of τ131 and the eWH domain of τ138 are included, see text for details. Additional predicted structural regions of τ131 and τ138 are highlighted in grey. HMG, high mobility group box domain; WH=winged helix. (**b**) Analytical size-exclusion chromatography profile of TFIIIC using a Superose 6 10/300 column (GE Healthcare). Known molecular weight standards at 670 and 158 kDa are indicated. Inset, Coomassie-stained SDS–PAGE gel of an elution peak fraction. (**c**) EMSA experiment of TFIIIC bound to a double-stranded (ds) 66 base-pair (bp) tDNA^Glu^ oligonucleotide. C, control (no TFIIIC added). (**d**) Crosslinking map of TFIIIC. TFIIIC subunits are represented as in **a** with internal vertical lines representing 100 amino-acid markers. Intra crosslinks are depicted by arcs that connect residues within the same subunit. Inter crosslinks are depicted by lines which connect residues within different subunits. Image produced using xiNET^50^.

As a first step in identifying the link between τA and τB subcomplexes, we performed a chemical crosslinking mass spectrometry (XL-MS) approach on purified, endogenous TFIIIC from *S. cerevisiae*. The protein complex was purified from a yeast strain, carrying a tandem affinity purification (TAP) tag on the τ60 subunit. Pure and stoichiometric TFIIIC could be obtained as assessed by size-exclusion chromatography and SDS–polyacrylamide gel electrophoresis (SDS–PAGE; Fig. 1b). The complex was shown to be functional by its ability to bind a double-stranded oligonucleotide containing A and B box sequences by electrophoretic mobility shift assay (EMSA; Fig. 1c). Using XL-MS, we identified 33 unique intersubunit and 89 unique intrasubunit crosslinks with high confidence at a linear discriminant (ld) score of>25 (Fig. 1d and Supplementary Tables 1 and 2). Twelve of the intersubunit crosslinks bridge τ131 with τ138. The majority of these links connect the N-terminal TPR array of τ131 to an unstructured, central region of τ138. With the exception of three additional crosslinks identified between the τ95 subunit and τ138, these links represent the major τA–τB connection revealed by our approach. Our XL-MS analyses also provide additional insights into the overall architecture of TFIIIC. Within the τA subcomplex, six crosslinks connect the dimerization domains of τ95 and τ55, consistent with our previously reported crystal structure^14^. In addition, we identified four crosslinks between the τ95 subunit and predicted TPRs at the C terminus of τ131. Within the τB subcomplex, there are surprisingly few crosslinks observed between the τ60 and τ91 subunits, despite the large interface between them^12^. Xlink Analyzer^33^ shows that some lysine residues are entirely buried in the τ60–τ91 interface and are therefore not accessible to the crosslinker. We also observe lysine residues surrounding this interface that are not detected by our XL-MS analysis, probably because they are buried in interfaces with other TFIIIC subunits. Four crosslinks connect τ91 with a disordered region between two predicted winged helix domains of τ138, which is consistent with the proposal of a co-operative role between τ91 and τ138 for B box binding^34^.

We went further in our analysis of the entire TFIIIC complex by performing XL-MS on DNA-bound TFIIIC, to determine whether the complex undergoes large conformational changes (Supplementary Tables 3 and 4). In general, we observed no fundamental changes in the intersubunit interaction network on tDNA binding (compare Fig. 1d and Supplementary Fig. 1). In particular, the bridge between the τ131 and τ138 subunits is maintained, indicating the importance of this link for the integrity and stability of TFIIIC when bound to DNA. Interestingly, the four crosslinks connecting the τ91 and τ138 subunits are not detected when TFIIIC is bound to DNA, suggesting a local change in conformation that prevents these previously crosslinked residues from accessing the crosslinker. It is tempting to speculate that this region is therefore involved in direct binding of TFIIIC to DNA.

### Structure of the τ131 TPR array

**Figure 2:**
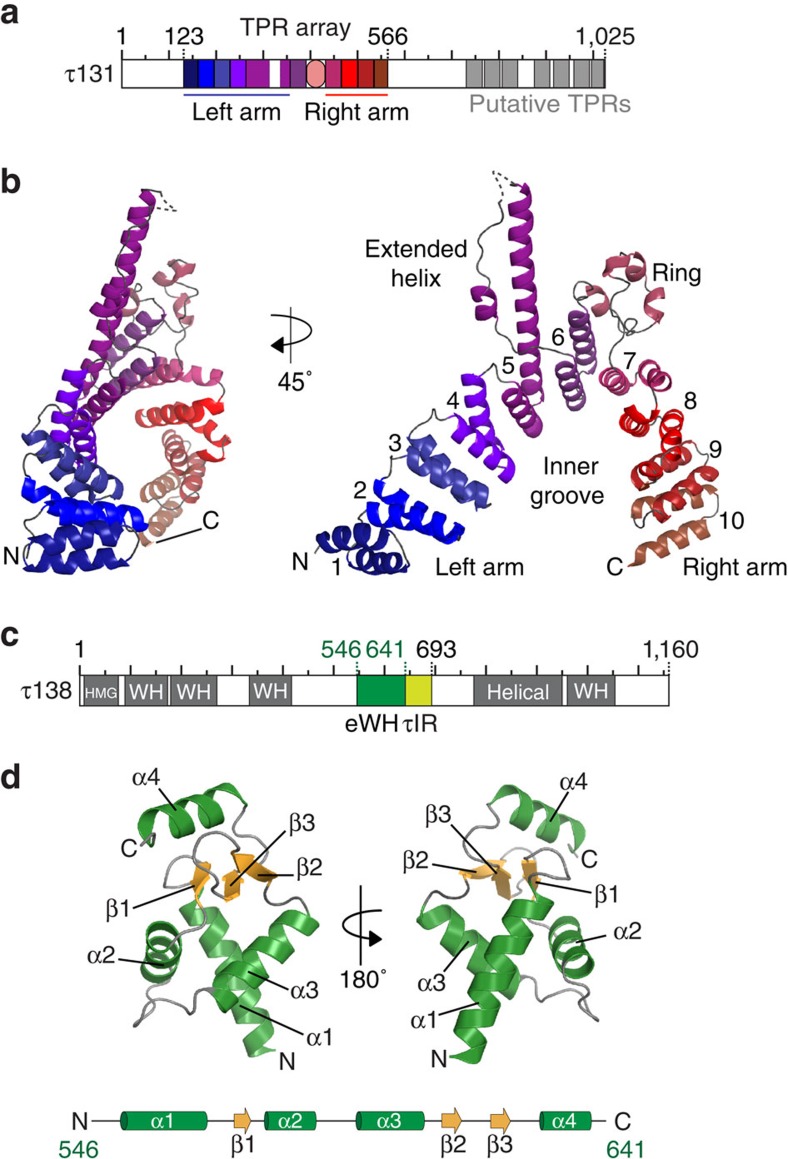
Crystal structures of the TPR array of τ131 and the central extended winged helix (eWH) domain of τ138. (**a**) Schematic domain architecture of τ131. The TPR array is highlighted and coloured according to the solved crystal structure in **b**. Putative TPRs are indicated in grey. (**b**) Crystal structure of the τ131 (123–566) TPR array in ribbon representation. Two views are displayed, related by a 45° rotation. A dashed line indicates a region of the electron density where no residues could be built with confidence (residues 317–336). TPRs are numbered 1–10. (**c**) Schematic domain architecture of τ138. Predicted winged helix (WH) domains, the high mobility group (HMG)-box domain and the helical region are shaded in grey. The central eWH domain is shaded in dark green. The τIR is shaded in light green (see text for details). (**d**) Crystal structure of the τ138 (546–641) eWH domain in ribbon representation. Two views are displayed, related by a 180° rotation. A schematic of the arrangement of α-helices and β-strands is displayed underneath the structure.

τ131 and τ138 are the largest subunits of the τA and τB subcomplexes, respectively^5^. τ131 was predicted to contain a highly conserved TPR array architecture at the N terminus with additional conserved TPRs predicted at the C terminus^19^ (Fig. 2a). Given that our crosslinking data implicated the TPR array of τ131 in connecting τA and τB, and the reported importance of this region for TFIIIB assembly, we determined the crystal structure of the TPR array (residues 123–566) in two different space groups (P6_2_ and P4_3_). Starting from a construct expressing residues 1–580 of τ131, we identified a stable fragment by limited proteolysis and mass spectrometry corresponding to residues 123–566. We expressed this truncated *S. cerevisiae* τ131 (123–566) protein in *E. coli*, purified it using affinity and size-exclusion chromatography and subsequently carried out crystallization trials. We were able to collect diffraction data up to 3.15 Å (P6_2_) and 3.4 Å (P4_3_) resolution. The lower-resolution structure was solved by multiple isomorphous replacement with anomalous signal (MIRAS), using anomalous signal from crystals prepared with selenomethionine substituted proteins or native crystals soaked with p-chloromercuribenzensulfonic acid to final *R*_work_/*R*_free_ values of 20.9%/24.5% The higher-resolution structure was solved using selenomethionine substituted protein in single anomalous dispersion (SAD) experiments and subsequently refined to *R*_work_/*R*_free_ values of 25.0%/28.7%. The combination of selenomethionine and mercury (Hg) markers aided the building of the structures. (Table 1, Supplementary Figs 2 and 3a and Supplementary Table 5).

The structure reveals the presence of 10 TPR repeats, while only nine TPR repeats had been predicted. Rather than forming an extended super-helix, the TPR array is instead separated into two ‘arms' by a central region that contains an extended helix and a disordered coil we call ‘ring' domain (Fig. 2b). This insertion causes a bend in the TPR array, positioning the TPRs of the right arm closer to the TPRs of the left arm. This feature generates a potential binding pocket we call the ‘inner groove'. τ131 is postulated as being highly flexible, consistent with its role in binding to multiple ligands at different stages in Pol III PIC assembly. A superimposition of the two crystal forms and an analysis of temperature factors reveal flexibility at TPRs 1–3, 8–10 and residues at the extremes of the extended helix (Supplementary Fig. 3b). Despite crystal contacts in these regions, the accommodation of the TPR array in these arrangements in two different crystal forms suggests that the conformational changes are indeed permitted within the TPR array.

### Characterization of the τ131–τ138 interaction region

**Figure 3:**
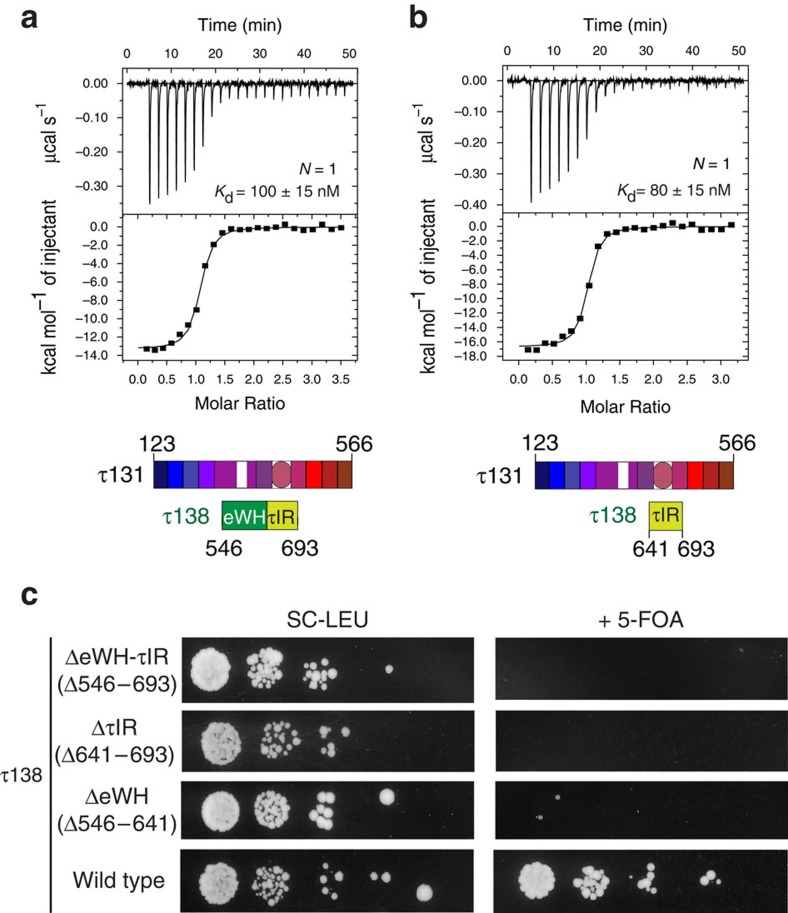
The τ131 TPR array interacts with high affinity to a central region of τ138. ITC measurement using purified (**a**) τ138 (546–693) and τ131 (123–566); (**b**) τ138 (641–693) and τ131 (123–566); Calculated *K*_d_ values and stoichiometry (N) are indicated. 15 μM of τ138 was used in the cell and 150 μM τ131 was used in the syringe in each case. (**c**) Viability of τ138 deletion mutants *in vivo* determined by the spot assay. A yeast strain carrying the plasmid pOL49 was transformed with the plasmids pRS415 ΔCEN τ138 and pRS415 ΔCEN Δ546-693 or Δ641-693 or Δ546-641 and plated on SC-URA-LEU medium. Serial 10-fold dilutions of all strains were spotted on SC-LEU and 5-fluoroorotic acid medium and were incubated for 72 h at 30 °C (*n*=3).

We next wanted to characterize further the central region of τ138 (546–693) and understand which regions were crucial for interaction with the TPR array. We hypothesized that the inner groove of the TPR array may accommodate the predicted central winged helix domain of τ138 (Fig. 2c). Surface analysis of the TPR array shows that the inner groove is lined with patches of conserved, often acidic surface residues that may bind to the predicted basic surface residues of τ138 (Supplementary Figs 3c,d,e and 4). We first solved the structure of the central domain of τ138 (546–641) using the sulphur-SAD technique (Table 1, Supplementary Table 5, Supplementary Fig. 5a). The 1.4 Å crystal structure (*R*_work_/*R*_free_ of 17.5%/19.8%) of this domain reveals a canonical winged helix domain that contains an additional C-terminal helix (Fig. 2d). This first structurally characterized part of τ138 thus represents an ‘extended' winged helix (eWH) domain^35^. The eWH domain is moderately well conserved from yeast to human (Supplementary Fig. 5b,c,d), and contains basic patches that could also suggest a role in binding nucleic acids (Supplementary Fig. 5e). However, we only detect very weak unspecific binding of the eWH domain to single and double-stranded DNA (Supplementary Fig. 5f). Isothermal titration calorimetry (ITC) did not detect an interaction between the eWH and the TPR array (Supplementary Fig. 6a). However, when we tested a construct that contained the eWH domain with additional residues at the C terminus (546–693), we observed a *K*_d_ for the interaction with the TPR array of ∼100 nM (Fig. 3a). A similar high-affinity interaction (∼80 nM) could be measured with only the unstructured region of τ138 (641–693) (Fig. 3b). Removal of residues 682–693 lowered the *K*_d_ of the interaction to ∼2.6 μM (Supplementary Fig. 6b), yet the region 641–681 was still essential to ensure the high-affinity interaction as tested peptides of 681–693 did not interact with the TPR array by ITC (data not shown). We thus concluded that the region 641–693 of τ138, hereafter referred to as ‘τ131-Interaction Region (τIR)', is necessary and sufficient to bind the TPR array of τ131.

To assess the *in vivo* importance of our *in vitro* results, we disrupted the chromosomal copy of τ138 in yeast and introduced a plasmid carrying wild-type τ138 and the URA3 gene under the control of the endogenous promoter, to maintain cell viability. This plasmid was then shuffled with a second plasmid carrying either the wild-type τ138 or a deletion mutant of τ138. We observed growth for cells transformed with the τ138 wild-type plasmid on FOA medium, but cells carrying the τ138 ΔeWH-τIR or the ΔτIR plasmid did not survive (Fig. 3c). This is a clear indication that these deletion mutants cannot complement the loss of wild-type τ138. Removal of residues 681–693 leads to an intermediate phenotype of reduced yeast growth (Supplementary Fig. 6c). Interestingly, the removal of just the eWH domain is also lethal to yeast (Fig. 3c), indicating that despite it being dispensable for the interaction with τ131 *in vitro*, it is still essential for proper TFIIIC function *in vivo*.

### τ138 and Bdp1 binding is affected by mutations in TPR 8

**Figure 4:**
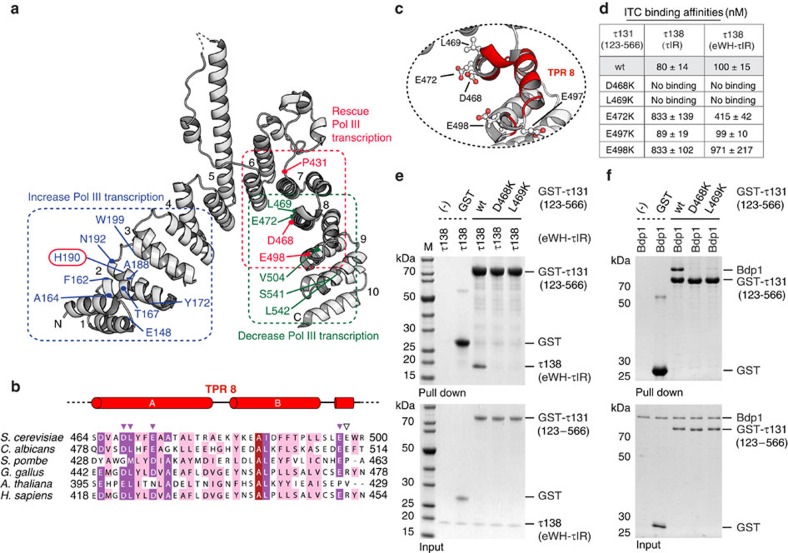
A binding hotspot on the τ131 TPR array for τ138 and Bdp1. (**a**) Mapped mutants of the τ131 TPR array (see text for details). (**b**) Sequence alignment of TPR 8. Identical residues are boxed in brick red, highly conserved in purple, medium conserved in pink and low conserved in white. Coloured arrowheads indicate the five mutated residues. (**c**) Close-up from the structure of TPR 8. The five residues selected for mutation are displayed as sticks; carbon atoms (grey); oxygen atoms (red). (**d**) Summary of ITC measurements using indicated τ131 (123–566) point mutants with τ138 (τIR) or τ138 (eWH-τIR). Wild-type (wt) measurements are included for reference. (**e**) GST pull-down assays of purified wild-type (wt) and mutant GST-tagged τ131 (123–566) variants with untagged τ138 (eWH-τIR). (−) indicates a background control for nonspecific binding of τ138 to the GST-affinity resin. A mixture of purified GST and untagged τ138 was also used as a negative control. Lower gel shows 5% of the input and upper gel shows bound fractions. (**f**) GST pull-down assays of purified wild-type (wt) and mutant GST-tagged τ131 (123–566) variants with untagged Bdp1. Negative controls and gel format as in **e**.

Our structure of the τ131 TPR array allows us, for the first time, to map and analyse mutations that have been previously described (Fig. 4a). In detail, mutations that increase Pol III transcription cluster mostly on TPR 2 (ref. 26), those that decrease Pol III transcription spread over TPRs 8–10 (ref. 22) and those that rescue a τ138 temperature-sensitive mutation, map mostly to TPRs 7–8 (ref. 16). Having mapped the critical τ131 interaction region of τ138 to ∼50 amino acids, we next questioned where the τIR binds on the TPR array. Despite extensive efforts, we were unable to obtain structural information of the τ131–τIR complex although both polypeptides form a stable complex during size-exclusion chromatography. Instead, we analysed previously described mutations together with the surface conservation of the TPR array (Supplementary Fig. 3e), to predict where the τIR may bind. We expressed and purified five τ131 point mutants that all cluster on TPR 8 (Fig. 4b). With the exception of residue L469, all of these residues are acidic and surface exposed (Fig. 4c). The purified mutants all eluted at the same volume from a size-exclusion column when compared with the wild type, indicating that the mutations did not cause a destabilization of the proteins (Supplementary Fig. 7a). Using ITC, we determined binding affinities of the mutant τ131 TPR arrays to the τIR and the eWH-τIR (Fig. 4d and Supplementary Fig. 7b). No binding of the mutants D468K and L469K to the τ138 proteins could be detected by ITC. Mutants E472K and E498K showed much weaker binding, while the E497K mutant showed no significant decrease in binding affinity to the τ138 proteins. These findings are consistent with results from GST pull-down assays (Fig. 4e and Supplementary Fig. 7c). We note that the L469K mutation likely causes a steric clash in the packing of TPR 8 with TPR 7 rather than abolishing a site-specific contact (Fig. 4c). Strikingly, the mutation of residues D468 and L469 has been previously implicated in a loss of binding to TFIIIB subunit Bdp1 (refs 16, 22). We purified full-length, recombinant Bdp1 and performed GST pull-down assays. Bdp1 binding to the TPR array of τ131 is strongly reduced in the D468K or L469K mutants (Fig. 4f). To our knowledge, this is the first time that a loss of interaction by both of these mutations has been probed directly using purified proteins. These results suggest that the binding hotspot for τ138 thus overlaps with that of a binding site for Bdp1.

### Brf1–TBP binds a distinct site from that of τ138 and Bdp1

Previous studies have proposed that τ131 exists in an auto-inhibited form before assembly of TFIIIB^27^. This auto-inhibition, possibly by the masking of the τ131 TPR array by the extended N terminus, may be relieved before or on recruitment of the Brf1 subunit of TFIIIB, paving the way for TBP and Bdp1 recruitment^28^. To further our understanding of the overlap between TFIIIB and τ138 binding, we purified a form of τ131 that contained the extended N terminus (1–580)^25^ as a GST fusion for interaction studies. As recombinant Brf1 is highly unstable, we were unable to produce sufficient quantities of pure, recombinant Brf1 protein for our studies. We therefore turned to a Brf1–TBP fusion protein that has been previously described and shown to functionally replace Brf1 both *in vitro* and *in vivo*^36^.

**Figure 5:**
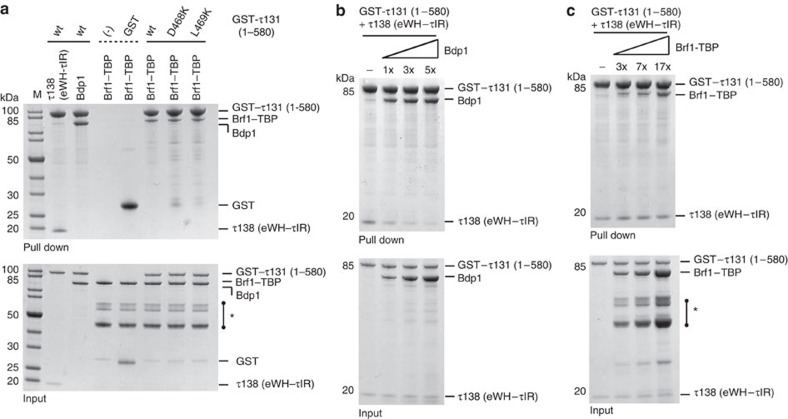
Defining the overlap between TFIIIB and τ138 binding to τ131. (**a**) GST pull-down assays of purified wild-type (wt) GST-tagged τ131 (1–580) with untagged τ138 (eWH-τIR) and Bdp1, and purified wild-type (wt) and mutant GST-tagged τ131 (1–580) variants with Brf1–TBP. (−) indicates a background control for nonspecific binding of Brf1–TBP to the GST-affinity resin. A mixture of purified GST and untagged Brf1–TBP was also used as a negative control. Lower gel shows 5% of the input and upper gel shows bound fractions. An * in the input gel indicates degradation products of Brf1–TBP. (**b**) GST pull-down competition assays of purified wild-type (wt) GST-tagged τ131 (1–580) with untagged τ138 (eWH-τIR) and Bdp1. GST-τ131 was preincubated with τ138 before addition of the indicated molar excess of Bdp1. (−) indicates a control experiment where no Bdp1 was added. Gel format as in **a**. (**c**) GST pull-down competition assays of purified wild-type (wt) GST-tagged τ131 (1–580) with untagged τ138 (eWH-τIR) and Brf1–TBP. GST-τ131 was preincubated with τ138 before addition of the indicated molar excess of Brf1–TBP. (−) indicates a control experiment where no Brf1–TBP was added. Gel format as in **a**. An * in the input gel indicates degradation products of Brf1–TBP.

We tested the binding of τ138 (eWH-τIR) and Bdp1 to τ131 (1–580) (Fig. 5a). Both proteins bind stoichiometrically to this longer τ131 protein, showing that the extended N terminus of τ131 does not inhibit binding. We did not observe binding of Brf1–TBP to τ131 (123–566) under our experimental conditions (data not shown); however, we did detect an interaction using τ131 (1–580; Fig. 5a). The interaction was also observed with the D468K and the L469K mutants of τ131 (1–580) (Fig. 5a). These results indicate that the extended N terminus of τ131 is required for high-affinity binding to Brf1–TBP, and that the principal binding site on τ131 is separate from the hotspot of τ138 and Bdp1. The purified Brf1–TBP contained degradation products of the fusion protein corresponding to truncations lacking the N terminus of Brf1, as confirmed by MS (Supplementary Fig. 8). We observed that these degradation products were not pulled down by τ131 (1–580; Fig. 5a), suggesting that the N terminus of Brf1, which includes the TFIIB-like cyclin repeats, is required for the interaction.

To conclusively determine if the τ138 binding site overlaps with Bdp1 and/or Brf1–TBP, we next used the GST-τ131 (1–580) and incubated it with τ138 (eWH-τIR), before titrating in increasing amounts of Bdp1 or Brf1–TBP. We observed a direct competition between τ138 and Bdp1 for binding to τ131 (Fig. 5b). These results were also replicated when using the shorter, TPR array of τ131 (data not shown). In contrast, even a ∼17-fold molar excess of Brf1–TBP could not compete out the τ138–τ131 interaction (Fig. 5c). Instead, both Brf1–TBP and τ138 can bind simultaneously to τ131, indicating that their binding sites are distinct.

## Discussion

**Figure 6:**
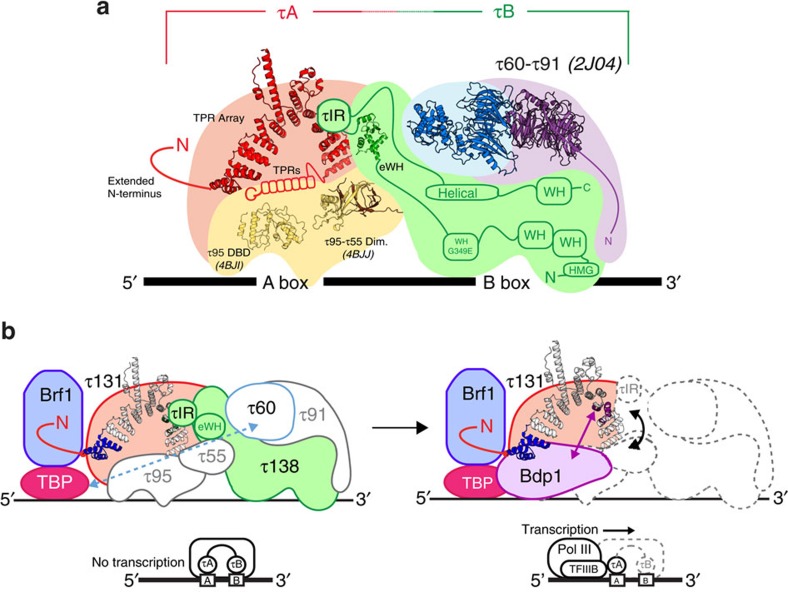
The role of τ131 in Pol III PIC formation. (**a**) Our current view of the arrangement of τA and τB subunits within TFIIIC based on interaction studies, crystal structures and crosslinking. Structures from this study are included, as well as previous structures with PDB codes indicated: the WD40 dimer structure of τ60–τ91 (ref. 12), and the τ95 DNA-binding domain (DBD)^14^ and τ95–τ55 dimerization homologues from *S. pombe*^14^. Note that the non-conserved τ55 histidine phosphatase domain (HPD) is omitted^13^. The extended N terminus and C-terminal TPRs of τ131 are indicated schematically. Predicted structural regions of τ138 are also indicated, including the G349E mutation. The disordered N terminus of τ91 is included schematically. (**b**) Model indicating two stages of PIC formation. In the first stage, recruitment of Brf1 to the PIC requires the extended N terminus of τ131 (red curve) and the N-terminal TPRs of the TPR array (highlighted in blue). TBP makes interactions with Brf1 and the τ60 subunit of τB. The link between τ131 and τ138 is maintained. In the second stage, the recruitment of Bdp1 involves conformational changes in the arms of the TPR array. τIR is displaced and the τA–τB link is altered, possibly leading to the disassembly of TFIIIC. TFIIIB is now assembled and recruits Pol III, together with τ131, for transcription.

On the basis of limited proteolysis and low-resolution scanning transmission light microscopy, TFIIIC has long been described as a ‘dumb-bell' shaped molecule consisting of two DNA-binding subcomplexes called τA and τB connected by a flexible linker^9^,^10^. More detailed information about the overall architecture of TFIIIC has been lacking. By combining structural information of individual subunits with our crosslinking data sets, we are able to provide a first model of the overall TFIIIC architecture (Fig. 6a). Our XL-MS data, combined with *in vitro* and *in vivo* mapping, indicate that the τIR establishes the main link between τA and τB, while the adjacent disordered regions on both sides of the eWH domain in τ138 presumably provide the necessary flexibility for binding variously spaced A box and B box promoters (Figs 2c and 6a). The N-terminal TPR array of τ131 provides a docking platform for the τIR, while C-terminal TPRs interact with the τ95 subunit of τA. Thus, τ131 is crucial for linking the τA and τB subunits. Subunits τ55 and τ95, the two other subunits of the τA subcomplex, share an unexpected structural similarity with the general transcription factor TFIIF^14^. Subunits τ55 and τ95 dimerize through a triple β-barrel domain consistent with several crosslinks that we observe between their dimerization domains, while the C-terminal DNA-binding domain of τ95 contains a winged helix domain similar to TFIIF Rap30 (ref. 14). In addition to the τIR, our XL-MS data reveal only one other possible link between τA and τB involving the dimerization domain of subunit τ95 and subunit τ138. The importance of this link will have to be substantiated in future studies. In τB, the WD40 propeller subunits τ60 and τ91 have been previously proposed to form a platform for τ138 interaction, thus cooperatively regulating B box binding of τ138 (refs 12, 34). We have observed crosslinks between the τ91 subunit and a disordered region between the third predicted winged helix domain and the eWH domain of τ138, which are not detected when TFIIIC is bound to DNA (Supplementary Fig. 1). In addition, it has been shown that a mutation in the third winged helix domain of τ138 (G349E) strongly reduces the affinity of TFIIIC for DNA^18^. Considering these different bodies of evidence it is tempting to speculate that this region in τ138 directly recognizes B box sequences.

In addition to advancing our understanding of TFIIIC architecture, our study also provides surprising insights into the overlap between τA, τB and TFIIIB interaction sites. Previous models have proposed that, due to its intrinsic flexibility, binding sites on the τ131 TPR array that are crucial for the binding of Brf1 and Bdp1 are masked in the context of the full TPR array and the extended N terminus^27^,^28^. This could be an important mode of regulation, preventing the assembly of TFIIIB until conditions are optimal for transcription to proceed. Our results provide new insights into the co-ordination and regulation of this assembly. We find that the extended N terminus of τ131 is absolutely required for high-affinity binding to Brf1–TBP. Under our experimental conditions, we were not able to detect an interaction between Brf1–TBP and the TPR array alone, although it seems certain that Brf1 can also bind here, as mutations that enhance Brf1 binding and stimulate Pol III transcription have been mapped to TPR 2 on the left arm of the array^26^ (Fig. 4a). Bdp1 can bind to τ131 without the presence of Brf1–TBP, and the extended N terminus of τ131 plays no role in inhibition of binding. Finally, our competition experiments have demonstrated that τ131 cannot accommodate τ138 and Bdp1 at the same time, whereas Brf1–TBP and τ138 can assemble simultaneously on τ131. We thus propose that τ138 prevents complete assembly of TFIIIB, with the crucial Bdp1 binding site masked by a disordered region of τ138, the τIR (Fig. 6b).

On the basis of previous studies and the results presented here, we propose the following sequential model for TFIIIB assembly—the assembly is initiated by the recruitment of Brf1 to τ131, most likely by the completed assembly of TFIIIC on a tRNA gene. The second TFIIIB component, namely TBP, is then recruited via binding sites on Brf1 and via the τB subunit τ60 (refs 12, 37). In the final step of TFIIIB assembly, Bdp1 is recruited to the TFIIIC/Brf1/TBP complex, competing with τ138 for a binding hotspot on TPR 8 of τ131. On binding, Bdp1 causes a conformational change or a break between τA and τB, displacing the critical τIR from the TPR array of τ131 and inducing the displacement of the τB module. TFIIIC (with the τB module providing most of the DNA-binding affinity) is only required for assembling TFIIIB but is dispensable for Pol III transcription^2^ and is displaced from its DNA-binding site during Pol III transcription as shown by EMSAs in an *in vitro* reconstituted system^38^. The mechanism by which *in vivo* TFIIIC is displaced (and presumably disassembled) during transcription is currently unknown. However, we suggest that Bdp1 could induce the displacement of the τB module as a regulatory mechanism essential for the initial round of Pol III transcription. Consistent with this model, a recent mass spectrometry study of TAP-purified Pol III from actively transcribing yeast cells detected co-eluting peptides from all three subunits of TFIIIB and peptides from the τ131 subunit, but not from any τB subunit^39^.

## Methods

### Purification of endogenous TFIIIC

TFIIIC was purified from *S. cerevisiae* strain SC2342 (provided by Cellzome AG), which expresses endogenous TFC8 (τ60) fused with a C-terminal TAP-tag. Yeast cells were grown overnight in YPD medium at 30 °C and 200 r.p.m. in a BIOSTAT C30 fermenter (Sartorius) under controlled conditions and collected at an OD_600 nm_ of 5–6. The cell paste was resuspended in lysis buffer (250 mM Tris-HCl, pH 8, 40% glycerol, 250 mM (NH_4_)_2_SO_4_, 1 mM EDTA, 12 mM β-mercaptoethanol) supplemented with protease inhibitors (Roche) before being lysed with glass beads in a BeadBeater (BioSpec). The lysate was centrifuged at 14,000 r.p.m. for 1 h at 4 °C, with the resulting supernatant then loaded onto a Heparin-Sepharose resin (GE Healthcare). The complex was eluted from the resin using high-salt buffer with 1 M (NH_4_)_2_SO_4_ and then diluted back to low salt for incubation with IgG Sepharose (GE Healthcare) for 6 h. After washing, IgG beads were incubated with TEV protease overnight at 4 °C. IgG-cleaved TFIIIC was recovered and subsequently purified by ionic exchange on a MonoQ column (GE Healthcare). TFIIIC was then applied to a Superose 6 10/300 column which had been pre-equilibrated in the final buffer (25 mM HEPES pH 7.5, 150 mM NaCl, 1 mM DTT). The eluted protein was subsequently concentrated to 1 μg μl^−1^.

### Chemical crosslinking of TFIIIC

Thirty μg (1 μg μl^−1^) of purified TFIIIC complex was crosslinked by addition of an iso-stoichiometric mixture of H12/D12 isotope-coded, di-succinimidyl-suberate (DSS, Creative Molecules). Equal amounts of crosslinker were added 10 times every 4 min to a final concentration of 2 mM. The crosslinking reactions were allowed to proceed for 40 min at 37 °C and quenched by the addition of ammonium bicarbonate to a final concentration of 50 mM for 10 min at 37 °C. Crosslinked proteins were denatured using urea and Rapigest (Waters) at a final concentration of 4 M and 0.05% (w/v), respectively. Samples were reduced using 10 mM DTT (30 min at 37 °C) and cysteines were carbamidomethylated with 15 mM iodoacetamide (30 min in the dark). Protein digestion was performed first using 1:100 (w/w) LysC (Wako Chemicals, Neuss, Germany) for 3.5 h at 37 °C then finalized with 1:50 (w/w) trypsin (Promega, Mannheim, Germany) overnight at 37 °C, after the urea concentration was diluted to 1.5 M. Samples were then acidified with 10% (v/v) TFA and desalted using MicroSpin columns (Harvard Apparatus). Crosslinked peptides were enriched using size-exclusion chromatography (SEC)^40^. In brief, desalted peptides were reconstituted with SEC buffer (30% (v/v) ACN in 0.1% (v/v) TFA) and fractionated using a Superdex Peptide PC 3.2/30 column (GE) on a Ettan LC system (GE Healthcare) at a flow rate of 50 ml min^−1^. Fractions eluting between 1 and 1.5 ml were evaporated to dryness and reconstituted in 50 μl 5% (v/v) ACN in 0.1% (v/v) FA.

### Mass spectrometry analysis of crosslinked peptides

Between 2% and 10% of the amount contained in the collected SEC fractions were analysed by liquid chromatography-coupled tandem mass spectrometry (MS/MS) using a nanoAcquity UPLC system (Waters) connected online to LTQ-Orbitrap Velos Pro instrument (Thermo). Peptides were separated on a BEH300 C18 (75 mm × 250 mm, 1.7 mm) nanoAcquity UPLC column (Waters) using a stepwise 60 min gradient between 3% and 85% (v/v) ACN in 0.1% (v/v) FA. Data acquisition was performed using a TOP-20 strategy where survey-MS scans (*m/z* range 375–1,600) were acquired in the Orbitrap (*R*=30,000) and up to 20 of the most abundant ions per full scan were fragmented by collision-induced dissociation (normalized collision energy=40, activation *Q*=0.250) and analysed in the LTQ. To focus the acquisition on larger crosslinked peptides, charge states 1, 2 and unknown were rejected. Dynamic exclusion was enabled with repeat count=1, exclusion duration=60 s, list size=500 and mass window±15 p.p.m. Ion target values were 1,000,000 (or 500 ms maximum fill time) for full scans and 10,000 (or 50 ms maximum fill time) for MS/MS scans. All the samples were analysed in technical duplicates. To assign the fragment ion spectra, raw files were converted to centroid mzXML using the Mass Matrix file converter tool and then searched using xQuest^41^ against a fasta database containing the sequences of the crosslinked proteins. Posterior probabilities were calculated using xProphet^41^ and results were filtered using the following parameters: false discovery rate=0.05, min delta score=0.95, MS1 tolerance window of 4 to 7 p.p.m., ld-score >25.

### Yeast strains

Plasmid pOL49 (a kind gift from O. Lefebvre) carrying a wild-type copy of τ138 (ref. 42) was transformed into a diploid yeast strain carrying a chromosomal deletion of τ138 (Euroscarf, Acc. Number Y20406). Haploid segregants carrying the τ138 deletion and the pOL49 plasmid were identified and isolated. τ138 was cloned by PCR amplification of genomic DNA, with primers spanning 500 bp up- and downstream of the coding sequence, and inserted into a plasmid carrying LEU selection (pRS415) to obtain pRS415-τ138. pRS415 is originally a CEN plasmid but since the genomic copy of τ138 carries a CEN sequence, this was removed from the pRS415-τ138 plasmid via restriction-free cloning^43^. The new plasmid was named pRS415 ΔCEN τ138. Restriction-free cloning was performed on plasmid pRS415 ΔCEN τ138 to create the deletion mutants.

### Cell viability—spot assays

Yeast strain carrying the plasmid pOL49 was transformed with the pRS415 ΔCEN τ138 plasmid set (including wild type, mutants and empty plasmid without τ138 gene) and plated on selective media (to select for both plasmids). Single colonies from fresh plates grown for 2 days at 30 °C were suspended in PBS to a final OD_600 nm_ of 0.4. Serial 10-fold dilutions of the different transformants were spotted on SC-LEU medium and 5-fluoroorotic acid containing medium, and incubated for 72 h at 30 °C. *n*=3.

### Protein expression and purification

The sequences for τ131 (123–566) and full-length Bdp1 were cloned into the pETM11 vector in frame with a TEV protease-cleavable N-terminal 6xHis tag and were transformed into BL21 Star (DE3) pRARE *E. coli* cells for expression. The codon-optimized sequence for τ138 (546–641) was cloned into the pETM30 vector in frame with a TEV protease-cleavable N-terminal 6xHis-GST tag and was transformed into BL21 (DE3) Gold *E. coli* cells for expression.

Cells were grown in TB till an OD_600 nm_ of 0.8, and protein expression was induced for 16 h at 18 °C with 0.5 mM isopropyl-β-D-thiogalactoside. Cells were harvested by centrifugation and resuspended in buffer A (5% glycerol, 50 mM Tris pH 7.5, 500 mM NaCl, 20 mM imidazole, 4 mM MgCl_2_, Complete EDTA-free Protease Inhibitor Cocktail (Roche), DNase1 (Roche), 2 mM β-mercaptoethanol) before being lysed by homogenization (Avestin Emulsiflex-C3). The lysate was centrifuged at 20,000 r.p.m. and the resulting supernatant was incubated with 2 ml Nickel-NTA agarose resin (Qiagen), preincubated in buffer B (50 mM Tris pH 7.5, 300 mM NaCl, 20 mM imidazole, 2 mM β-mercaptoethanol), for 1 h at 4 °C. The resin was applied to a disposable column and washed with 20 column volumes of buffer B, 20 column volumes of buffer B containing 1 M NaCl and again with 20 column volumes of buffer B. The protein was eluted off the column using buffer B containing 250 mM imidazole and incubated with TEV protease for 16 h at 4 °C in buffer B. Cleaved protein was reapplied to the resin and collected in the flow-through, before being applied to a pre-equilibrated (20 mM Tris pH 7.5, 150 mM NaCl, 2 mM DTT) preparative S200 26/60 column (GE Healthcare). Cloning of the τ131 point mutants was carried out using protocols and reagents provided in the Quikchange Lightning kit (Agilent). Mutants were purified by the same method as the wild type.

### Protein expression and purification of Brf1–TBP

The sequence of the Brf1–TBP fusion protein Brf1 (1–382)-TBP (61–240)-Brf1 (439–596) has been described previously^36^. The sequence was cloned into the pETM13 vector in frame with a non-cleavable C-terminal 6xHis tag. The vector was transformed into BL21 Star (DE3) pRARE *E. coli* cells and grown in TB till an OD_600 nm_ of 0.8. Protein expression was induced for 16 h at 18 °C with 0.5 mM isopropyl-β-D-thiogalactoside. Lysis and histidine-affinity purification was as described above, but with 5% glycerol added to all ‘B' buffers. Eluted protein was diluted in buffer C (5% glycerol, 50 mM Tris pH 7.5, 2 mM DTT) so that the final NaCl concentration was 150 mM. The sample was applied to a 5 ml HiTrap SP HP column (GE Healthcare) that had been pre-equilibrated in buffer D (5% glycerol, 50 mM Tris pH 7.5, 150 mM NaCl, 2 mM DTT). The column was washed with 20 column volumes of buffer D, before a gradient of 40 column volumes into buffer E (5% glycerol, 50 mM Tris pH 7.5, 1 M NaCl, 2 mM DTT) was applied.

### Crystallization and X-ray structure determination

For τ131 (123–566), crystals of the P4_3_ and P6_2_ space group were grown at 20 °C by the hanging-drop vapour diffusion method at a concentration of 49 mg ml^−1^ and 60 mg ml^−1^ respectively, with a 1:1 ratio of protein and crystallization solutions. For P4_3_, the crystallization solution contained 0.1 M bicine pH 8.9 and 0.85 M MgCl_2_. Crystals were cryo-protected by soaking in mother liquor containing 30% glycerol before being flash-frozen in liquid nitrogen. For P6_2_, the crystallization solution contained 0.2 M MgCl_2_, 0.1 M Tris pH 8.3 and 42.5% ethylene glycol. Crystals were flash-frozen in liquid nitrogen using the already present ethylene glycol as a cryo-protectant. X-ray data for native, selenomethionine-incorporated and Hg crystals were collected at the ESRF and PETRAIII beamlines. The data was processed with X-ray diffuse scattering (XDS)^44^. The P4_3_ crystals were also consistently indexed with pointless and each data set was further scaled with SCALA. Derivatives were scaled to the native data set with scaleit. The P6_2_ structure was solved by SAD combined with density modification using the programme autoSHARP^45^. The P4_3_ structure was solved by MIRAS combined with density modification using autoSHARP. For both structures, iterative model building and refinement was carried out using Coot^46^ and Phenix^47^, respectively. The initial models were used as molecular replacements models for native, higher-resolution data sets using PHASER^48^. The final models were validated using MolProbity^49^.

For τ138 (546–641), crystals were grown at 20 °C by the hanging-drop vapour diffusion method at a concentration of 30 mg ml^−1^. Protein solution and crystallization solution were mixed in a 1:1 ratio. The crystallization solution contained 1.15 M Na citrate pH 6.2 and 0.1 M Na cacodylate. Crystals were cryo-protected by soaking in mother liquor containing 15% glycerol before being flash-frozen in liquid nitrogen. Data were collected on an in-house rotating anode to record single-wavelength anomalous signal from sulphur atoms. The data were processed using XDS. The initial substructure, based on anomalous signal from sulphur atoms, was solved using autoSHARP. Six sulphur sites were identified, indicating two molecules in the asymmetric unit. Phasing equations were solved and density modification was performed using autoSharp. Automatic building of an initial model was carried out using AutoBuild in Phenix. A higher-resolution native data set on a second crystal was collected at the ID23-1 beamline at the ESRF, and was processed using XDS. The structure was solved by molecular replacement with Phaser using the initial model from the sulphur-SAD experiment above. Iterative model building and refinement was then carried out using Coot and Phenix respectively. The final model was validated using MolProbity.

### GST pull-down experiments

GST-tagged τ131 and τ138 proteins were expressed in BL21 (DE3) Gold *E. coli* cells, and purified with a GST preparative FF 16/10 column (GE Healthcare) followed by size-exclusion chromatography using a pre-equilibrated (20 mM Tris pH 7.5, 150 mM NaCl, 2 mM DTT) S200 26/60 column (GE Healthcare). For pull-down experiments, 15 μg of GST-tagged τ131 or τ138 protein and their tested binding partners were incubated with 25 μl Glutathione Sepharose 4B beads in buffer F (50 mM Tris pH 7.5, 150 mM NaCl, 2 mM DTT, 0.1% Tween20) at 4 °C for 4 h. τ138 (300 nM) and Bdp1, and 1 μM of Brf1–TBP were used when testing binding to the GST-τ131 constructs. Wild-type (900 nM) and mutant τ131 was used when testing the binding to GST-τ138 (641–693). After incubation, the beads were washed three times with buffer F, before being heated to 100 °C in Laemmli sample buffer for 5 min.

### ITC experiments

ITC was performed using a MicroCal ITC200 System (GE Healthcare). All samples were dialyzed into ITC buffer (20 mM Tris pH 7.5, 150 mM NaCl, 2 mM β-mercaptoethanol). Protein concentration in the cell and syringe was 15 and 150 μM, respectively. Experiments were performed at 25 °C.

### EMSA of TFIIIC and τ138 (546–641)

The sequence of the 66 base-pair oligonucleotide (5′-CGA TAT AGT GTA ACG GCT ATC ACA TCA CGC TTT CAC CGT GGA GAC CGG GGT TCG ACT CCC CGT ATC-3′) contains A and B box elements from a tDNA^Glu^ sequence (underlined). HPLC-purified oligonucleotides were ^32^P end-labelled by T4 Polynucleotide Kinase before subsequent gel purification by denaturing 15% urea-PAGE. To form double-stranded oligonucleotides, the ^32^P-labelled oligonucleotides were heated to 95 °C for 2 min, cooled to 25 °C and incubated in EMSA buffer (20 mM Tris pH 7.5, 150 mM NaCl, 1 mM MgCl_2_, 2 mM DTT). For the EMSA, single-stranded or double-stranded oligonucleotides were incubated with TFIIIC or τ138 (546–641) at 25 °C for 30 min, before being run at 120 V on a 4.5% acrylamide gel in Tris-glycine buffer. The gel was dried and autoradiographed with X-ray film (Biomax, MR-film, Kodak).

## 

## Additional information

**Accession codes**: The coordinates and structure factors of the τ131 TPR array in space group P43 and P62 (PDB 5AEM and 5AIO) and τ138 eWH (PDB 5AIM) have been deposited with the European Protein Data Bank.

**How to cite this article:** Male, G. *et al.* Architecture of TFIIIC and its role in RNA polymerase III pre-initiation complex assembly. *Nat. Commun.* 6:7387 doi: 10.1038/ncomms8387 (2015).

## Supplementary Material

Supplementary Information Supplementary Figures 1-8 and Supplementary Tables 1-5

## Figures and Tables

**Table 1:** Data collection and refinement statistics.

	**τ131 Native P6**_**2**_	**τ131 Native P4**_**3**_	**τ138 Native**
*Data collection*			
Beamline	PETRAIII (P14)	ESRF (ID23-2)	ESRF (ID23-1)
Space group	P6_2_	P4_3_	H32
Cell dimensions			
*a=b*, *c* (Å)	116.36 95.98	105.13 98.74	129.09 68.04
Wavelength (Å)	0.97626	0.87260	0.9763
Resolution (Å)*,†	50–3.15 (3.23–3.15)	73.34–3.4 (3.58–3.4)	50–1.4 (1.44–1.4)
CC ½ (%)	0.99 (0.49)	0.99 (0.68)	0.99 (0.65)
R_merge_ (%)	7.4 (275.3)	8.2 (58.7)	5.1 (137.9)
*I*/*σI*	34.41 (1.7)	8.6 (2.0)	21.3 (1.7)
Completeness (%)	99.9 (99.9)	99.9 (99.3)	99.9 (99.3)
Redundancy sites	39.3 (38.3)	4.0 (3.5)	10.1 (9.4)
			
*Refinement*			
Resolution (Å)	50–3.15	74.36–3.4	50–1.4
No. of reflections‡	12,884	14,867	42,553
*R*_work_/*R*_free_ (%)	25.00/28.66	20.92/24.45	17.52/19.75
No. of non-H atoms			
Protein	3,442	3,379	1,581
Ligand/ion	0	0	12
Water	0	0	197
B-factors (Å^2^)			
Protein	151.1	145.6	32.3
Ligand/ion	0	0	67.8
Water	0	0	38.0
r.m.s deviations			
Bond lengths (Å)	0.004	0.006	0.006
Bond angles (°)	0.713	1.1	0.947

r.m.s., root mean squared.

^*^Values in parentheses correspond to the highest-resolution shell.

^†^Resolution cutoff criteria according to ref. 51. Resolution limits according to *I*/*σ* of 2 are 3.2 Å for τ131 in P6_2_, 3.4 Å for τ131 in P4_3_, 1.45 Å for τ138, respectively.

^‡^Non-anomalous, anomalous: 28,928; number of reflections in *R*_free_ set is 1,468.
