# Supplementary material for: Architecture of TFIIIC and its role in RNA polymerase III pre-initiation complex assembly
Source: Nat Commun. 2015 Jun 10;6:7387. doi: 10.1038/ncomms8387 (PMC4490372; doi:10.1038/ncomms8387)
Supplement: Supplementary Information — Supplementary Figures 1-8 and Supplementary Tables 1-5 [file ncomms8387-s1.pdf]

Supplementary Information

Architecture of TFIIIC and its role in RNA polymerase III pre-initiation complex assembly

Gary Male, Alexander von Appen, Sebastian Glatt, Nicholas M. I. Taylor, Michele Cristovao, Helga Groetsch, Martin Beck and Christoph W. Müller

Supplementary Figures

Supplementary Figure 1: Cross-linking mass spectrometry of DNA-bound TFIIIC

Supplementary Figure 2: Electron densities for the τ131 TPR array crystal structures

Supplementary Figure 3: Analyzing the crystal structure of the TPR array of τ131

Supplementary Figure 4: Sequence and structural alignment of the TPR array of τ131

Supplementary Figure 5: Analyzing the crystal structure and DNA-binding capability of the eWH domain of τ138

Supplementary Figure 6: Mapping the τ131-τ138 interaction *in vitro* and *in vivo*

Supplementary Figure 7: Determining effects of τ131 point mutations on τ138 interaction

Supplementary Figure 8: Mass spectrometry analysis of Brf1-TBP degradation products

Supplementary Tables

Supplementary Table 1: TFIIIC Inter-subunit cross-links

Supplementary Table 2: TFIIIC Intra-subunit cross-links

Supplementary Table 3: DNA-bound TFIIIC Inter-subunit cross-links

Supplementary Table 4: DNA-bound TFIIIC Intra-subunit cross-links

Supplementary Table 5: Data collection statistics

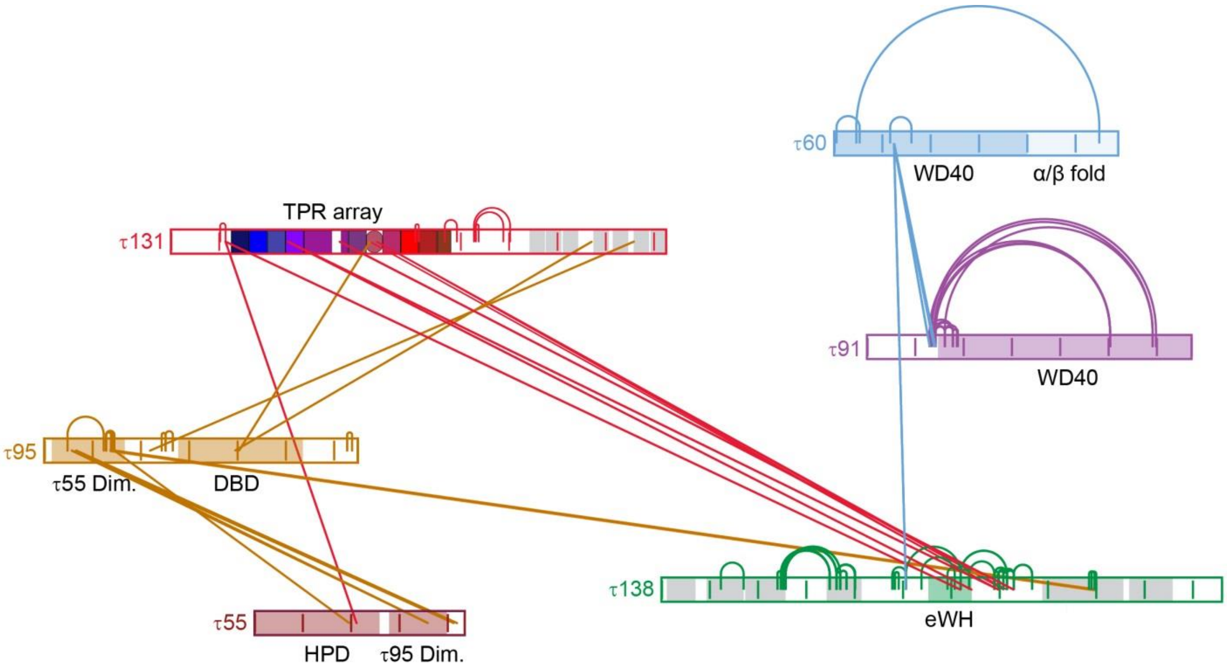

Supplementary Figure 1: Cross-linking mass spectrometry of DNA-bound TFIIIC. Cross-linking map of DNA-bound TFIIIC. TFIIIC was pre-incubated with a 66 base-pair double-stranded oligonucleotide (see Online Methods). TFIIIC subunits are represented as bars with internal vertical lines representing 100 amino acid markers. Intra cross-links are depicted by arcs which connect residues within the same subunit. Inter cross-links are depicted by lines connecting different subunits. Domains of which crystal structures are available are highlighted. τ55 and τ95 Dim. = τ55 and τ95 dimerisation domains; DBD = DNA-binding domain; HPD = histidine phosphatase domain. The TPR array of τ131 and the extended winged helix domain (eWH) of τ138 are also highlighted. Additional predicted structural domains are highlighted in grey (see also Fig.1 (a) and (d)).

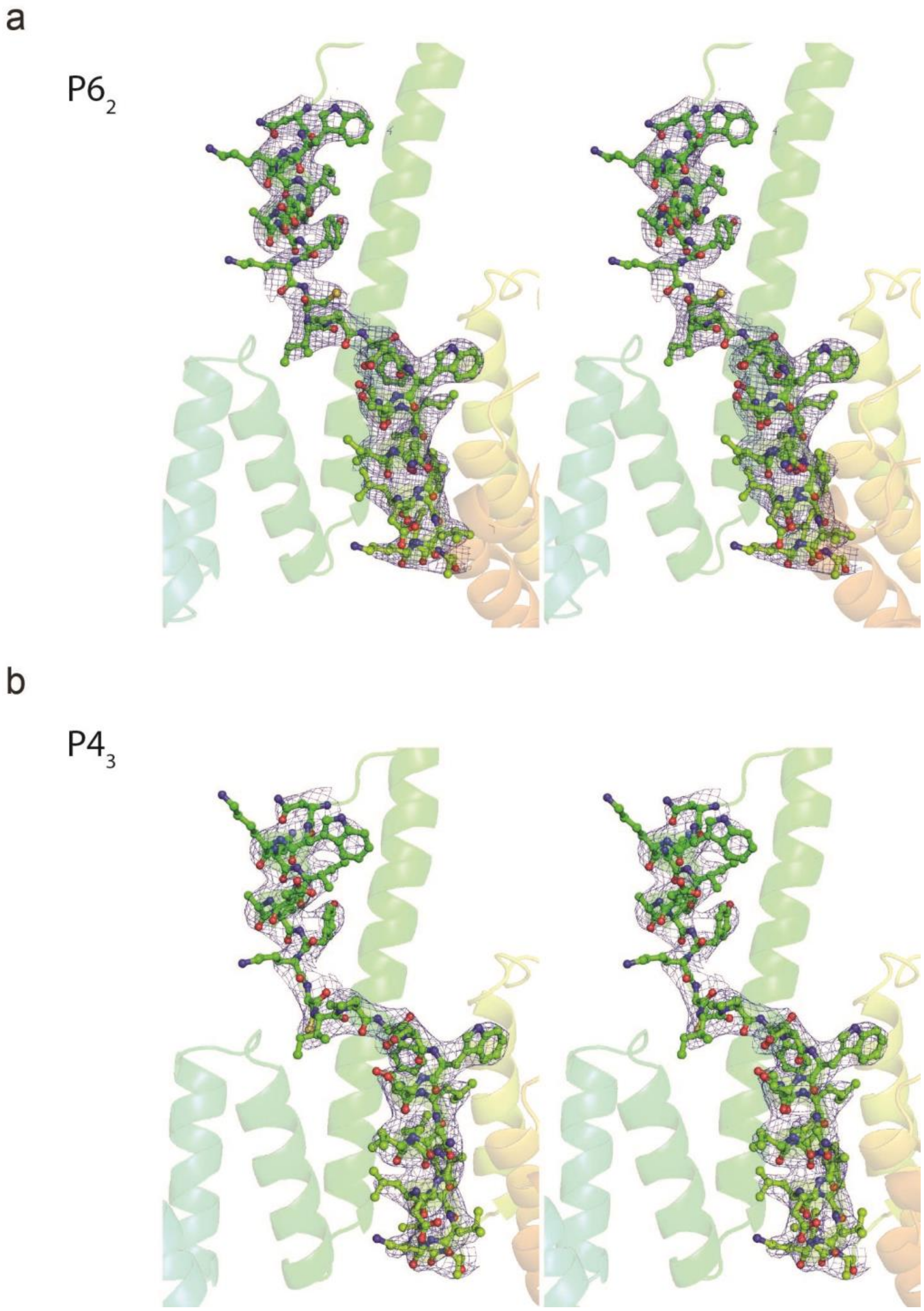

Supplementary Figure 2: Electron densities of the τ131 TPR array crystal structures. (a) Stereo images of a portion of the refined electron density (2mFo-dFc) at 1σ contour level for the τ131 TPR array crystal structure in space group $P6_2$ at 3.15 Å resolution. (b) The same region is depicted in space group $P4_3$ at 3.4 Å resolution.

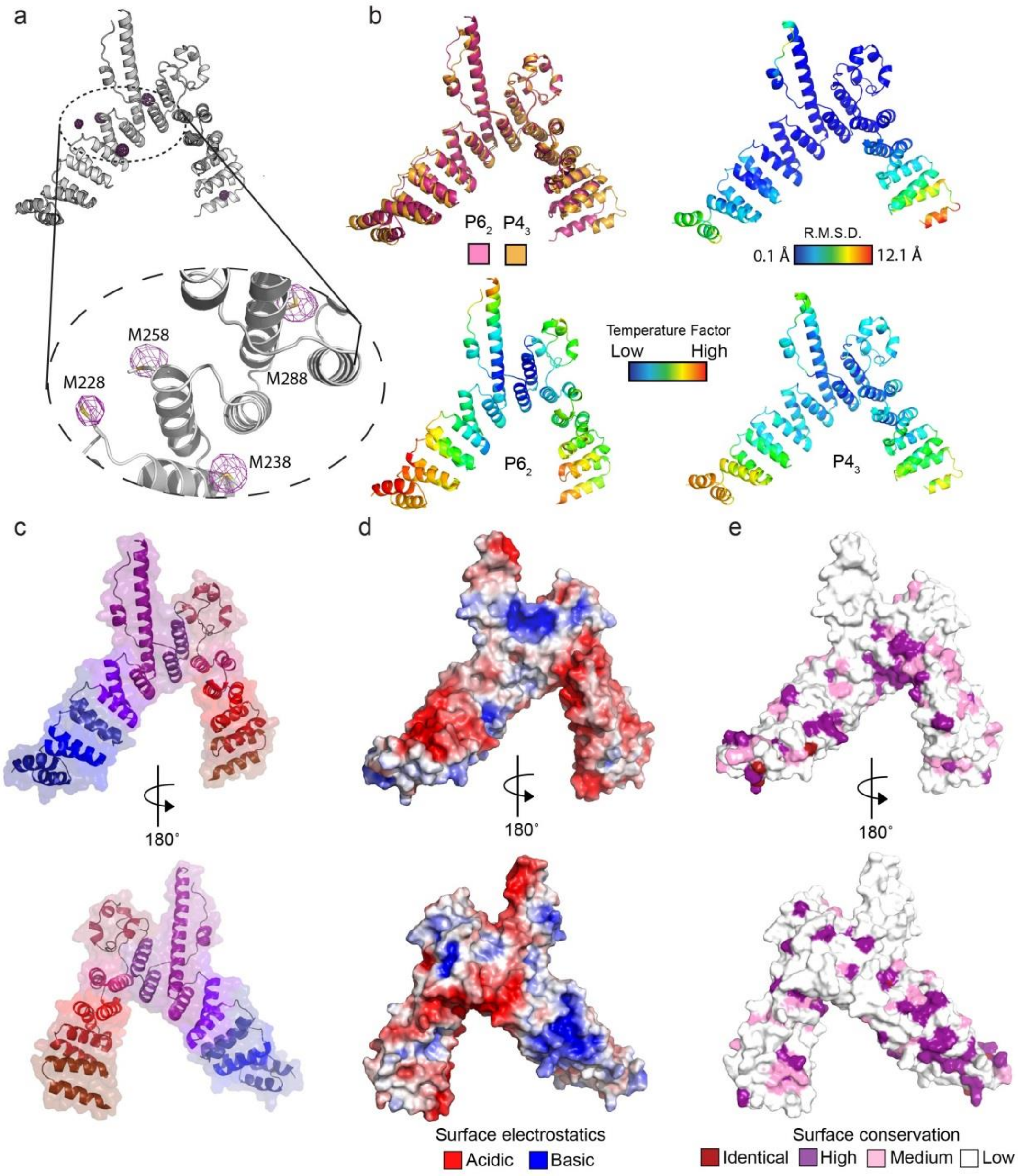

Supplementary Figure 3: Analyzing the crystal structure of the TPR array of τ131. (a) Anomalous difference Fourier map (purple) showing selenomethionine positions contoured at 5σ. (b) *Top left*: Superimposition of the two crystal forms of the TPR array. The overall, sequence-matched r.m.s.d.$_{407C\alpha}$ = 2.99 Å. *Top right*: Heat map of the r.m.s.d. between the two crystal forms. *Bottom:* The TPR array structures colored according to temperature factors. (c) Two views of the TPR array in ribbon representation with a semi-transparent surface representation overlaid. (d) Surface electrostatic charge representations of the corresponding views in (c). (e) Surface conservation representations of the corresponding views in (c).

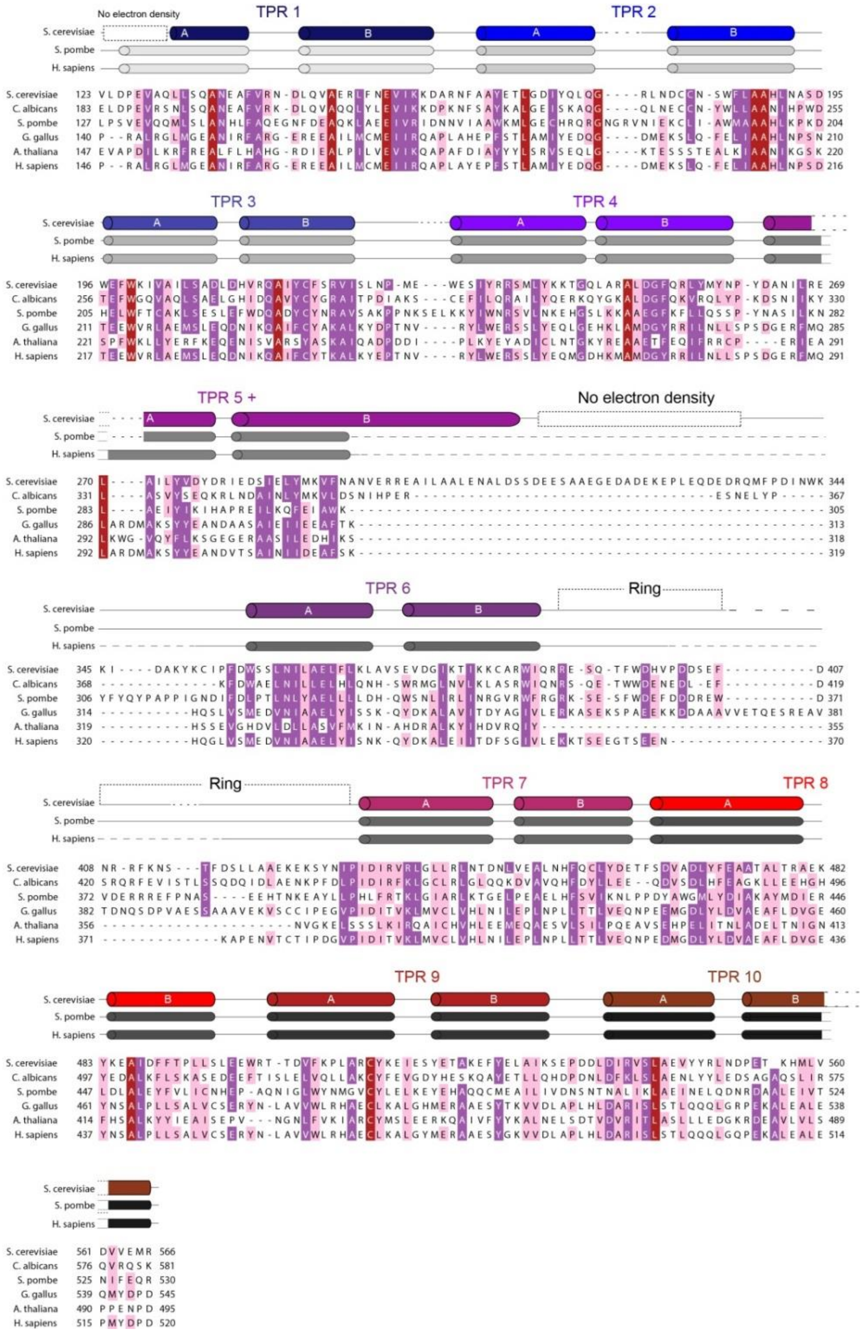

Supplementary Figure 4: Sequence and structural alignment of the TPR array of τ131. Alignment of the *S. cerevisiae* TPR array sequence with corresponding TPR array sequences from other species. Identical residues are boxed in brick red, highly conserved residues in purple, medium conserved residues in pink and low conserved residues in white. A structural alignment of predicted TPR array structures from *S. pombe* and *H. sapiens* with the *S. cerevisiae* TPR array is also indicated.

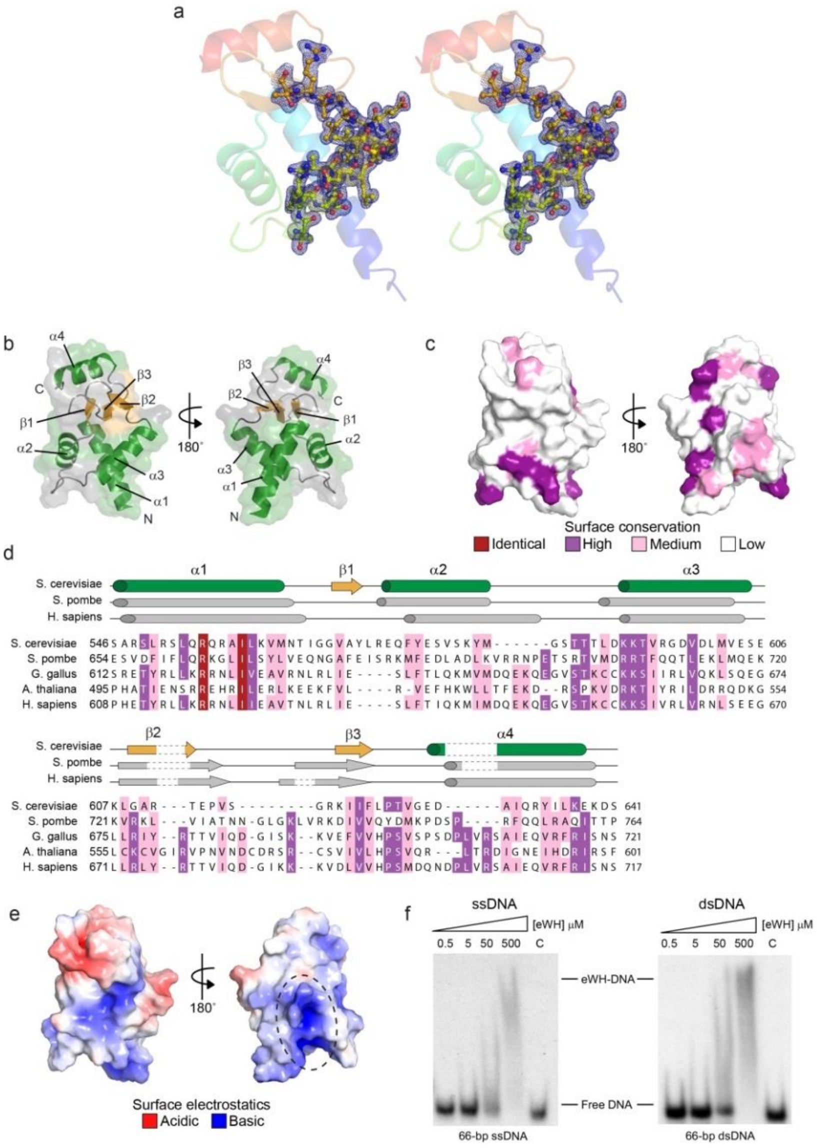

Supplementary Figure 5: Analyzing the crystal structure and DNA-binding capability of the eWH domain of τ138 (546-641). (a) Stereo image of a portion of the refined electron density (2mFo-dFc) at 1σ contour level. (b) Two views of the eWH domain in ribbon representation with a semi-transparent surface representation overlaid. (c) Surface conservation representations of the corresponding views in (a). (d) Alignment of the *S. cerevisiae* eWH domain sequence with corresponding sequences from other species. (e) Surface electrostatic charge representations of the corresponding views in (a). The conserved basic surface batch mainly formed by basic residues R557 and K561 is highlighted. (f) EMSA experiments of the eWH domain bound to a single-stranded (ss) or double-stranded (ds) 66 base-pair (bp) oligonucleotide. C = control DNA reaction (no eWH domain added).

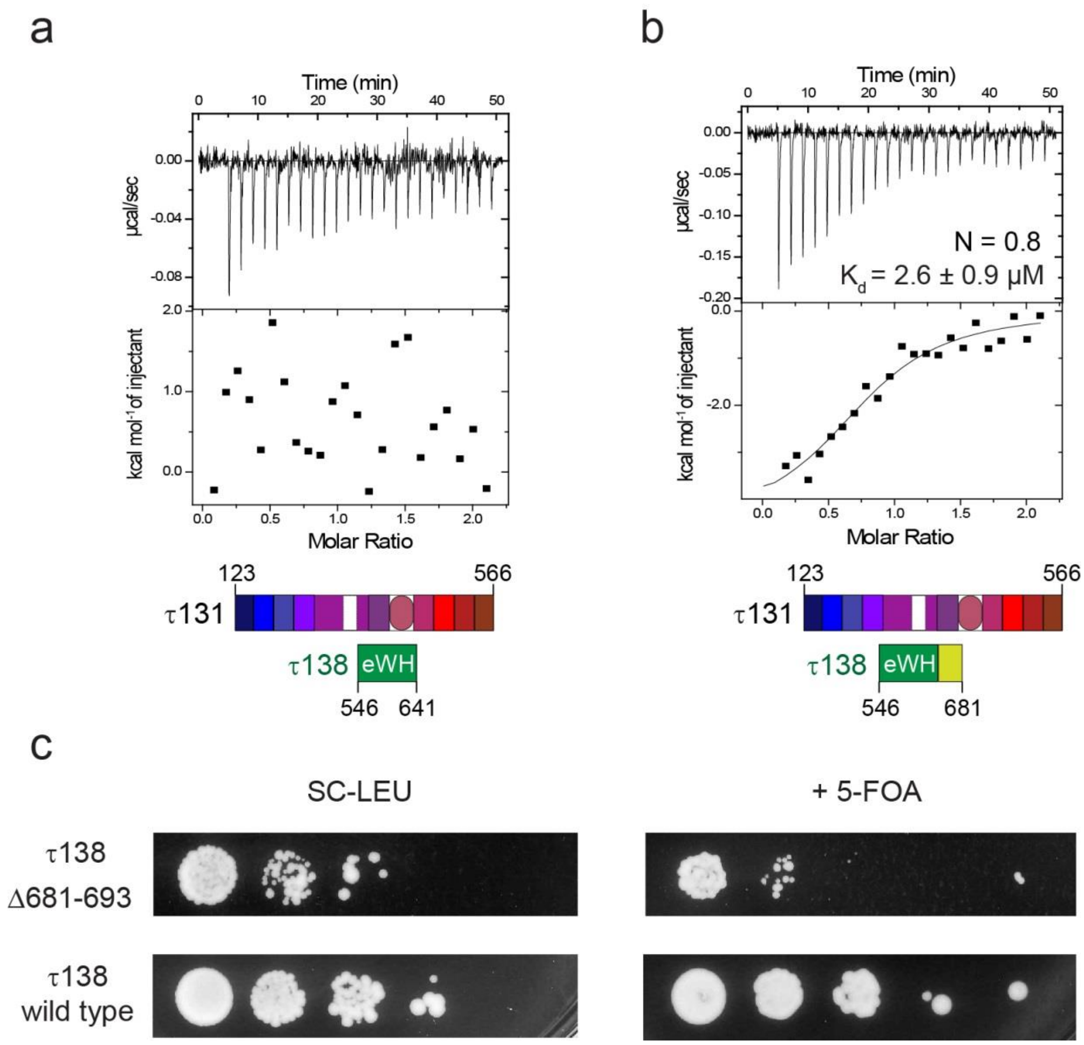

Supplementary Figure 6: Mapping the τ131-τ138 interaction in vitro and in vivo. ITC measurement using purified: (a) τ138 (546-641) and τ131 (123-566); (b) τ138 (546-681) and τ131 (123-566); Calculated Kd values and stoichiometry (N) are indicated. 15 µM of τ138 was used in the cell and 150 µM τ131 was used in the syringe in each case. (c) Viability of τ138 Δ681-693 in vivo determined by the spot assay (n=3).

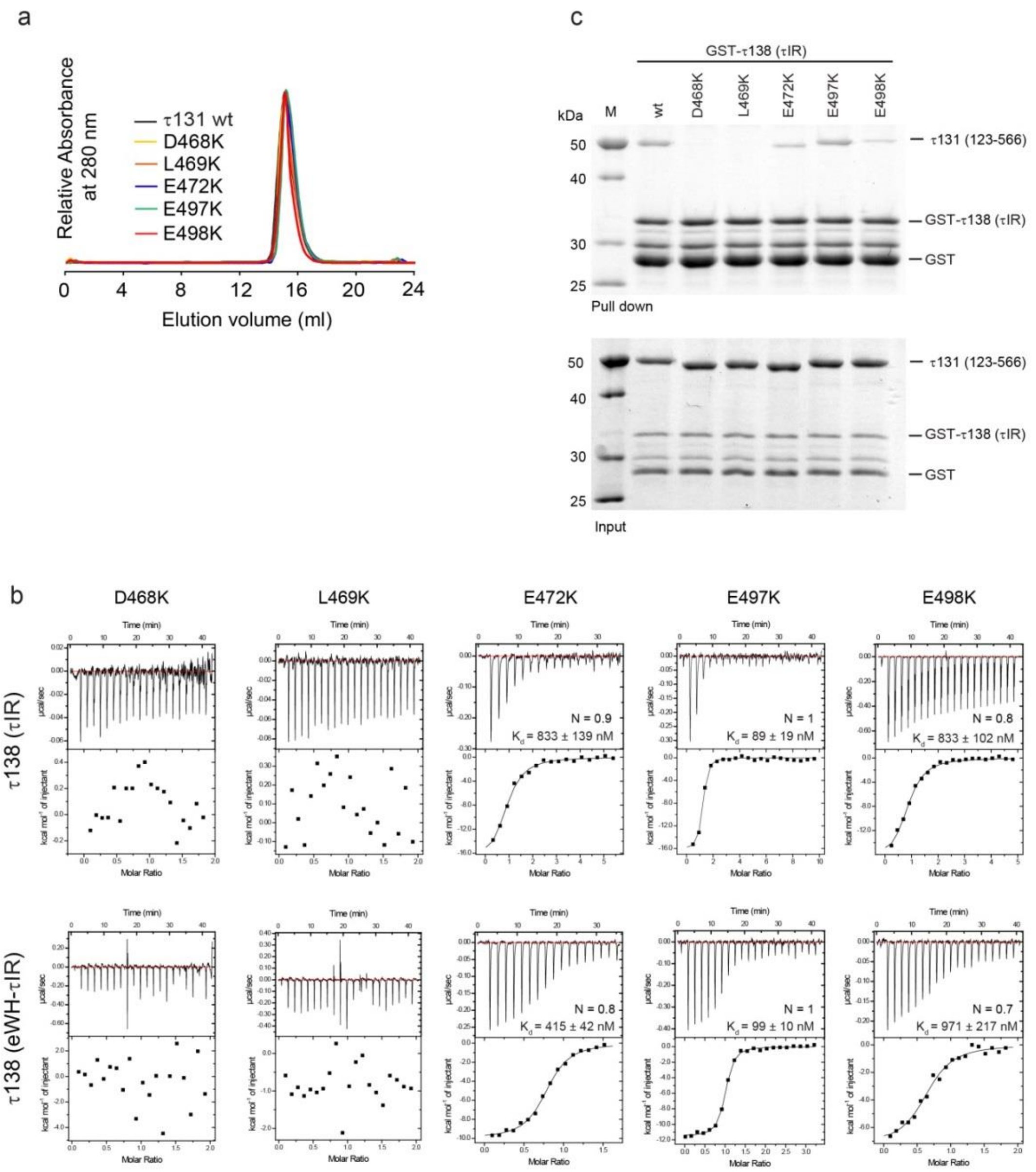

Supplementary Figure 7: Determining effects of τ131 point mutations on τ138 interaction. (a) Analytical gel filtration of τ131 (123-566) wildtype (wt) and mutants using a Superose 6 10/300 column (GE Healthcare). (b) ITC measurements using purified τ138 (τIR) or τ138 (eWH-τIR) (15 μM in the cell) and τ131 (123-566) mutants (150 μM in the syringe). Calculated K_d values and stoichiometry (N) are indicated. (c) GST-pull down assays of purified wild type (wt) GST-tagged τ138 (τIR) with untagged τ131 (123-566) wildtype (wt) and point mutants. Lower gel shows 5% of the input and upper gel shows bound fractions.

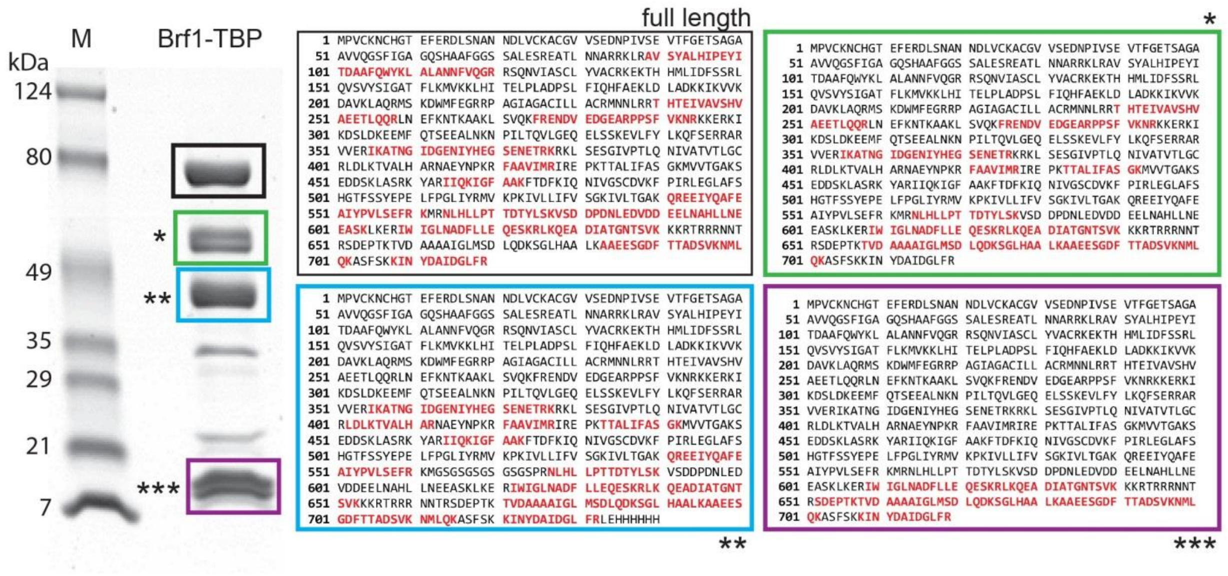

Supplementary Figure 8: Mass spectrometry analysis of Brf1-TBP degradation products. *Left*: SDS-PAGE of the purified Brf1-TBP fusion protein. *Right*: Results from the MASCOT analyses of the indicated gel bands. Identified peptides are highlighted in red with the sequence of the expressed Brf1-TBP fusion protein. Bands and corresponding MS results are labelled by colour and asterisks.

Supplementary Table 1: TFIIIC Inter-subunit cross-links

Peptide*	Protein 1	Protein 2	Lysine 1	Lysine 2	ld-score**
MLTNLDS**K**GNLSSR-NM**K**SSSPGSSLGQK	τ60	τ91	124	142	46.39
ISAST**K**DLSK-DSI**K**SLK	τ138	τ95	896	138	43.28
ISAST**K**DLSK-SL**K**DSNK	τ138	τ95	896	141	40.11
**K**STSVNVSDGK-**K**TGQLAR	τ138	τ131	698	242	39.36
MLTNLDS**K**GNLSSR-NST**K**NMK	τ60	τ91	124	139	39.35
**K**TAGLLSPTEENETTNAGQSK-V**K**EAFK	τ55	τ95	359	66	38.54
ASDDAIS**K**K-**K**TGQLAR	τ138	τ131	697	242	38.51
DHTGDKEEVQSE**K**IYR-V**K**EAFK	τ55	τ95	419	66	38.33
NSSATQ**K**SLR-GFDELG**K**SR	τ91	τ138	598	433	37.53
F**K**NSTFDSLLAAEK-I**K**ASDDAISK	τ131	τ138	412	689	36.91
MLTNLDS**K**GNLSSR-NTVQ**K**LR	τ60	τ91	124	130	35.78
E**K**SYNIPIDIR-I**K**ASDDAISK	τ131	τ138	426	689	35.28
DHTGD**K**EEVQSEK-AI**K**MCGGIEK	τ55	τ95	412	57	34.5
DHTGD**K**EEVQSEK-V**K**EAFK	τ55	τ95	412	66	30.13
**K**VLVVSPK-YEKM**K**K	τ138	τ131	240	636	28.79
**K**LLSSPIVK-EVYNPN**K**K	τ95	τ131	395	871	28.67
E**K**SYNIPIDIR-**K**STSVNVSDGK	τ131	τ138	426	698	28.39
F**K**NSTFDSLLAAEK-ASDDAIS**K**K	τ131	τ138	412	697	28.37
SSSPGSSLGQ**K**GRPIR-MLTNLDS**K**GNLSSR	τ91	τ60	153	124	28.36
E**K**SYNIPIDIR-ASDDAIS**K**K	τ131	τ138	426	697	28.33

DHTGDKEEVQSE**<u>K</u>**IYR-VTMP**<u>K</u>**GTLSK	τ55	τ95	419	123	27.86
DESVIL**<u>K</u>**VTMPK-S**<u>K</u>**NGIR	τ95	τ138	118	200	27.62
LDP**<u>K</u>**TAETMK-YH**<u>K</u>**IRK	τ95	τ131	631	947	27.25
LFTAQNFQ**<u>K</u>**LTNAEDEISVPK-WDYSI**<u>K</u>**DDKYR	τ138	τ91	414	610	27.09
RI**<u>K</u>**LEQHVSTAQEPK-NSSATQ**<u>K</u>**SLR	τ138	τ91	509	598	26.67
**<u>K</u>**TGQLAR-**<u>K</u>**VSAGR	τ131	τ138	242	715	26.17
TTVVVENT**<u>K</u>**EDK-KIDA**<u>K</u>**YK	τ131	τ138	729	349	26.15
MSLNENGD**<u>K</u>**IR-DSN**<u>K</u>**LR	τ55	τ95	196	145	26.08
AIL**<u>K</u>**VMNTIGGVAYLR-**<u>K</u>**TGQLAR	τ138	τ131	561	242	25.72
IYGLN**<u>K</u>**EK-IPPNS**<u>K</u>**K	τ91	τ138	178	478	25.69
LLSSPIV**<u>K</u>**K-EVYNPN**<u>K</u>**K	τ95	τ131	403	871	25.54
SLYTDLE**<u>K</u>**QEADYNLGR-SV**<u>K</u>**IPSTDFQLPPPPK	τ131	τ95	958	219	25.4
ITA**<u>K</u>**VVDK-**<u>K</u>**TYTRK	τ131	τ138	627	885	25.24

* Cross-linked lysine residues are underlined and in bold

** Peptides are listed in descending order of ld Score, only cross-links with an ld score > 25 were selected for analysis

Supplementary Table 2: TFIIIC Intra-subunit cross-links

Peptide*	Protein 1	Protein 2	Lysine 1	Lysine 2	ld-score**
ITG**K**EFQR-NPAI**K**IR	τ138	τ138	362	252	45.76
**K**VLVVSPK-NPAI**K**IR	τ138	τ138	240	252	41.79
LL**K**DLSSAR-NST**K**NMK	τ91	τ91	161	139	41.38
IYGLN**K**EK-LLLLA**K**VK	τ91	τ91	178	186	40.74
LLHGI**K**NSSATQK-LL**K**DLSSAR	τ91	τ91	591	161	40.34
**K**STSVNVSDGK-I**K**ASDDAISK	τ138	τ138	698	689	39.79
ITA**K**VVDKYEK-**K**HQVDETLHR	τ131	τ131	627	567	39.12
SGL**K**GISTMDVVNR-NPAI**K**IR	τ138	τ138	348	252	39.12
I**K**ASDDAISK-**K**VSAGR	τ138	τ138	689	715	39.1
NM**K**SSSPGSSLGQK-LL**K**DLSSAR	τ91	τ91	142	161	38.13
AFT**K**SSEYYLESVDK-ITG**K**EFQR	τ138	τ138	370	362	38.05
GDVDLMVESE**K**LGAR-G**K**VVNFGGFSAR-	τ138	τ138	607	538	37.99
LL**K**DLSSAR-LLLLA**K**VK	τ91	τ91	161	186	37.61
ITG**K**EFQR-CV**K**YVK	τ138	τ138	362	257	37.52
A**K**IDAHGINITCTK-WDYSI**K**DDKYR	τ91	τ91	634	610	37.26
NEIEAL**K**NVGNESIDNVIMDMAK-VPLG**K**PFSR	τ138	τ138	930	1073	37.18
NM**K**SSSPGSSLGQK-LLHGI**K**NSSATQK-	τ91	τ91	142	591	36.96
DINCNS**K**NLFHVK-LL**K**DLLVDRK	τ60	τ60	53	5	36.7
TTVVVENT**K**EDK-**K**VSAGR	τ138	τ138	729	715	36.63
ISAST**K**DLSK-**K**TYTRK	τ138	τ138	896	885	36.61
LL**K**DLSSAR-IYGLN**K**EK	τ91	τ91	161	178	36.47
DLS**K**SQSDDYIR-VPLG**K**PFSR	τ138	τ138	900	1073	36.04

TAETM**K**SELK-IA**K**LDPK	τ95	τ95	637	627	35.94
GTLS**K**NNNSVK-DSI**K**SLK	τ95	τ95	128	138	35.7
SAEDSPSSNGGTVV**K**GK-GDVDLMVESE**K**LGAR	τ138	τ138	536	607	35.69
IPSLELPLNVST**K**HSSIQK-VTMP**K**GTLSK	τ95	τ95	48	123	35.51
LL**K**DLSSAR-NTVQ**K**LR	τ91	τ91	161	130	35.27
SSNDTSDISSKPLLEDS**K**FR-**K**HQVDETLHR	τ131	τ131	599	567	34.86
NSSATQ**K**SLR-NTVQ**K**LR	τ91	τ91	598	130	34.18
KSTSVNVSDG**K**IK-**K**VSAGR	τ138	τ138	708	715	33.99
CY**K**EIESYETAK-**K**FVGILR	τ131	τ131	513	678	33.83
CY**K**EIESYETAK-TTDVF**K**PLAR	τ131	τ131	513	506	33.47
IYDFEG**K**KK-CV**K**YVK	τ138	τ138	400	257	33.4
IT**K**LFPNNSLDNLK-TTVVVENT**K**EDK	τ138	τ138	767	729	33.32
NST**K**NMK-NTVQ**K**LR	τ91	τ91	139	130	33.11
STSVNVSDG**K**IK-ASDDAIS**K**K	τ138	τ138	708	697	32.98
I**K**ASDDAISKK-EKDS**K**K	τ138	τ138	689	642	32.85
SSNDTSDISS**K**PLLEDSKFR-**K**HQVDETLHR	τ131	τ131	592	567	32.74
NM**K**SSSPGSSLGQK-DIATT**K**TTVSR	τ91	τ91	142	503	32.68
SSSPGSSLGQ**K**GRPIR-NST**K**NMK	τ138	τ138	153	139	32.57
SSSPGSSLGQ**K**GRPIR-NSSATQ**K**SLR	τ91	τ91	153	598	32.49
**K**HQVDETLHR-VVD**K**YEK	τ131	τ131	567	631	32.39
**K**STSVNVSDGK-**K**VSAGR	τ138	τ138	698	715	32.31
**K**FNTELDFQIER-VVD**K**YEK	τ131	τ131	688	631	32.27
SSSPGSSLGQ**K**GRPIR-NTVQ**K**LR	τ91	τ91	153	130	32.26

SSNDTSDISSKPLLEDS**K**FR-**K**RTPYDAER	τ131	τ131	599	607	32.09
NM**K**SSSPGSSLGQK-RNTVQ**K**LR	τ91	τ91	142	130	32.04
V**K**EGFETSVFDFPFK-IYGLN**K**EK	τ91	τ91	188	178	32.03
YA**K**DINCNSK-ICPVS**K**QR	τ60	τ60	46	549	32.01
EASNF**K**VK-G**K**SYGR	τ131	τ131	102	113	31.85
TDL**K**TLNEDNFVALNNTVR-RS**K**NGIR	τ138	τ138	439	200	31.73
EFEDW**K**NNLTWAR-**K**ITDLK	τ60	τ60	18	228	31.67
AFT**K**SSEYYLESVDK-CV**K**YVK	τ138	τ138	370	257	31.4
SSSPGSSLGQ**K**GRPIR-DIATT**K**TTVSR	τ91	τ91	153	503	31.33
RLLHGI**K**NSSATQK-NST**K**NMK	τ91	τ91	591	139	31.16
RLLHGI**K**NSSATQK-NTVQ**K**LR	τ91	τ91	591	130	30.94
I**K**ASDDAISKK-YIL**K**EK	τ138	τ138	689	637	30.94
LS**K**LAEGDSVFEGPLMEER-ITA**K**VVDKYEK	τ131	τ131	702	627	30.8
NSTNASVAGNISNP**K**R-I**K**LEQHVSTAQEPK-	τ138	τ138	506	509	30.78
IPSLELPLNVST**K**HSSIQK-AI**K**MCGGIEK	τ95	τ95	48	57	30.59
RQV**K**NSTNASVAGNISNPK-RI**K**LEQHVSTAQEPK	τ138	τ138	491	509	30.26
ISNE**K**SSNDTSDISSKPLLEDSK-**K**HQVDETLHR	τ131	τ131	581	567	30.18
NSTNASVAGNISNP**K**R-G**K**VVNFGGFSAR	τ138	τ138	506	538	29.71
AFT**K**SSEYYLESVDK-GHVV**K**QLK	τ138	τ138	370	165	29.51
DN**K**MLTNLDSK-VIDI**K**R	τ60	τ60	116	556	29.31
IPPNS**K**K-TPN**K**NK	τ138	τ138	478	483	29.28

TTVVVENTKED**K**TVYHAGTK-**K**VSAGR	τ138	τ138	732	715	29.28
EI**K**RPLFLYK-**K**FTLVR	τ138	τ138	839	854	29.26
SAEDSPSSNGGTVV**K**GK-AIL**K**VMNTIGGVAYLR	τ138	τ138	536	561	29.22
DIATT**K**TTVSR-NTVQ**K**LR	τ91	τ91	503	130	29.12
IYDFEG**K**K-CV**K**YVK	τ138	τ138	400	257	29.1
AFT**K**SSEYYLESVDK-Q**K**ENTGGYR	τ138	τ138	370	383	28.95
LS**K**LAEGDSVFEGPLMEER-VVD**K**YEK	τ131	τ131	702	631	28.9
IT**K**LFPNNSLDNLK-**K**VSAGR	τ138	τ138	767	715	28.82
LFTAQNFQ**K**LTNAEDEISVPK-Q**K**ENTGGYR	τ138	τ138	414	383	28.4
NM**K**SSSPGSSLGQK-NSSATQ**K**SLR	τ91	τ91	142	598	27.99
**K**FELNSGLNEAK-VVD**K**YEK	τ131	τ131	637	631	27.81
NEIEAL**K**NVGNESIDNVIMDMAK-DLS**K**SQSDDYIR	τ138	τ138	930	900	27.7
ASDDAIS**K**K-**K**VSAGR	τ138	τ138	697	715	27.63
SSSPGSSLGQ**K**GRPIR-LL**K**DLSSAR	τ91	τ91	153	161	27.34
SGE**K**GINTMDLAQVTGQDPR-**K**INHLLTSSQLIYK	τ138	τ138	123	147	27.31
NVP**K**PPPLVFESDTPGGIDSR-KLLSSPIV**K**K	τ95	τ95	408	403	27.29
ANPFA**K**KK-**K**NGVTEVK	τ95	τ95	251	254	27.08
EASNF**K**VK-**K**NDKGK	τ131	τ131	102	108	26.84
GTLS**K**NNNSVK-SL**K**DSNK	τ95	τ95	128	141	26.59
**K**STSVNVSDGK-E**K**DSKK	τ138	τ138	698	639	26.34
GTLS**K**NNNSVK-DSN**K**LR	τ95	τ95	128	145	26.15

NM**K**SSSPGSSLGQK-IYGLN**K**EK	τ91	τ91	142	178	26.09
Q**K**ENTGGYR-IPPNS**K**K	τ138	τ138	383	478	25.69

* Cross-linked lysine residues are underlined and in bold

** Peptides are listed in descending order of ld Score, only cross-links with an ld score > 25 were selected for analysis

Supplementary Table 3: DNA-bound TFIIIC Inter-subunit cross-links

Peptide*	Protein 1	Protein 2	Lysine 1	Lysine 2	ld-score**
MLTNLDS**K**GNLSSR-NTVQ**K**LR	τ60	τ91	124	130	42.62
MLTNLDS**K**GNLSSR-NM**K**SSSPGSSLGQK	τ60	τ91	124	142	40.62
ISAST**K**DLSK-DSI**K**SLK	τ138	τ95	896	138	39.75
MLTNLDS**K**GNLSSR-NST**K**NMK	τ60	τ91	124	139	39.49
NSTNASVAGNISNP**K**R-MLTNLDS**K**GNLSSR	τ138	τ60	506	124	38.69
**K**IIFLPTVGEDAIQR-G**K**SYGR	τ138	τ131	619	113	36.55
**K**TAGLLSPTEENETTNAGQSK-V**K**EAFK	τ55	τ95	359	66	35.82
SGSCSLDKYEIL**K**K-G**K**SYGR	τ55	τ131	211	113	35.04
E**K**SYNIPIDIR-**K**STSVNVSDGK	τ131	τ138	426	698	34.8
**K**LLSSPIVKK-EVYNPN**K**K	τ95	τ131	395	871	34.77
KISAST**K**DLSK-SL**K**DSNK	τ138	τ95	896	141	33.85
E**K**SYNIPIDIR-ASDDAIS**K**K	τ131	τ138	426	697	33.8
F**K**NSTFDSLLAAEK-ASDDAIS**K**K	τ131	τ138	412	697	33.77
DHTGDKEEVQSE**K**IYR-V**K**EAFK	τ55	τ95	419	66	32.98
DHTGD**K**EEVQSEK-AI**K**MCGGIEK	τ55	τ95	412	57	31.98
**K**STSVNVSDGK-**K**TGQLAR	τ138	τ131	698	242	30.83
**K**TAGLLSPTEENETTNAGQSK-AI**K**MCGGIEK-	τ55	τ95	359	57	30.05
MSLNENGD**K**IR-DSN**K**LR	τ55	τ95	196	145	29.03
E**K**SYNIPIDIR-**K**VSAGR	τ131	τ138	426	715	28.93

SLYTDLE**<u>K</u>**QEADYNLGR-SV**<u>K</u>**IPSTDFQLPPPPK	τ131	τ95	958	219	28.7
ASDDAIS**<u>K</u>**K-**<u>K</u>**TGQLAR	τ138	τ131	697	242	27.44
TTVVVENT**<u>K</u>**EDK-KIDA**<u>K</u>**YK	τ138	τ131	729	349	27.41
F**<u>K</u>**NSTFDSLLAAEK-LLSSPIV**<u>K</u>**K	τ131	τ95	412	403	27.12
STSVNVSDG**<u>K</u>**IK-E**<u>K</u>**SYNIPIDIR	τ138	τ131	708	426	26.64
**<u>K</u>**TGQLAR-EKDS**<u>K</u>**K	τ131	τ138	242	642	25.82
**<u>K</u>**TGQLAR-**<u>K</u>**VSAGR	τ131	τ138	242	715	25.04

* Cross-linked lysine residues are underlined and in bold

** Peptides are listed in descending order of ld Score, only cross-links with an ld score > 25 were selected for analysis

Supplementary Table 4: DNA-bound TFIIIC Intra-subunit cross-links

Peptide*	Protein 1	Protein 2	Lysine 1	Lysine 2	ld-score**
NM**K**SSSPGSSLGQK-IYGLN**K**EK	τ91	τ91	142	178	44.25
NM**K**SSSPGSSLGQK-LL**K**DLSSAR	τ91	τ91	142	161	43.22
GTLS**K**NNNSVKDSIK-DSN**K**LR	τ95	τ95	128	145	41.4
**K**NSENTPEFYFESSIR-DN**K**MLTNLDSK	τ60	τ60	160	116	41.27
**K**VLVVSPK-NPAI**K**IR	τ138	τ138	240	252	41.23
SGL**K**GISTMDVVNR-NPAI**K**IR	τ138	τ138	348	252	40.52
GDVDLMVESE**K**LGAR-G**K**VVNFGGFSAR	τ138	τ138	607	538	40.42
LLHGI**K**NSSATQK-LL**K**DLSSAR	τ91	τ91	591	161	39.97
LL**K**DLSSAR-IYGLN**K**EK	τ91	τ91	161	178	39.91
ITG**K**EFQR-CV**K**YVK	τ138	τ138	362	257	39.8
NM**K**SSSPGSSLGQK-DIATT**K**TTVSR	τ91	τ91	142	503	39.66
GTLS**K**NNNSVK-DSI**K**SLK	τ95	τ95	128	138	39.23
ISAST**K**DLSK-**K**TYTRK	τ138	τ138	896	885	38.7
**K**STSVNVSDGK-I**K**ASDDAISK	τ138	τ138	698	689	38.35
ASDDAIS**K**K-**K**VSAGR	τ138	τ138	697	715	38.33
DIATT**K**TTVSR-NTVQ**K**LR	τ91	τ91	503	130	38.15
IYGLN**K**EK-LLLLA**K**VK	τ91	τ91	178	186	38.13
GTLS**K**NNNSVK-SL**K**DSNK	τ95	τ95	128	141	38.11
NST**K**NMK-NTVQ**K**LR	τ91	τ91	139	130	38.1
NM**K**SSSPGSSLGQK-NTVQ**K**LR	τ91	τ91	142	130	38.01
DINCNS**K**NLFHVK-LL**K**DLLVDRK	τ60	τ60	53	5	37.14
TAETM**K**SELK-IA**K**LDPK	τ95	τ95	637	627	36.38

NSTNASVAGNISNP**K**R-**K**IIFLPTVGEDAIQR	τ138	τ138	506	619	35.91
I**K**ASDDAISK-**K**VSAGR	τ138	τ138	689	715	35.84
LLHGI**K**NSSATQK-NST**K**NMK	τ91	τ91	591	139	35.43
V**K**EGFETSVFDFPFK-IYGLN**K**EK	τ91	τ91	188	178	35.37
**K**FELNSGLNEAK-VVD**K**YEK	τ131	τ131	637	631	35.16
**K**IIFLPTVGEDAIQR-YIL**K**EK	τ138	τ138	619	637	35.1
AFT**K**SSEYYLESVDK-CV**K**YVK	τ138	τ138	370	257	34.92
**K**FNTELDFQIER-VVD**K**YEK	τ131	τ131	688	631	34.86
TTVVVENT**K**EDK-**K**VSAGR	τ138	τ138	729	715	34.78
DLS**K**SQSDDYIR-**K**ISASTK	τ138	τ138	900	890	34.68
SGL**K**GISTMDVVNR-VLVVSP**K**NPAIK	τ138	τ138	348	247	34.65
YA**K**DINCNSK-ICPVS**K**QR	τ60	τ60	46	549	34.63
**K**IIFLPTVGEDAIQR-**K**VSAGR	τ138	τ138	619	715	34.61
CY**K**EIESYETAK-TTDVF**K**PLAR	τ131	τ131	513	506	34.46
LL**K**DLSSAR-NST**K**NMK	τ91	τ91	161	139	34.18
STSVNVSDG**K**IK-ASDDAIS**K**K	τ138	τ138	708	697	34.14
**K**STSVNVSDGK-**K**VSAGR	τ138	τ138	698	715	33.99
AFT**K**SSEYYLESVDK-ITG**K**EFQR	τ138	τ138	370	362	33.94
IYGLN**K**EK-NST**K**NMK	τ91	τ91	178	139	33.84
IT**K**LFPNNSLDNLK-TTVVVENT**K**EDK	τ138	τ138	767	729	33.81
SSNDTSDISS**K**PLLEDSK-**K**HQVDETLHR	τ131	τ131	592	567	33.4
I**K**ASDDAISK-YIL**K**EK	τ138	τ138	689	637	33.27
IPSLELPLNVST**K**HSSIQK-VTMP**K**GTLSK	τ95	τ95	48	123	33.08
STSVNVSDG**K**IK-**K**VSAGR	τ138	τ138	708	715	32.98

DIATT**K**TTVSR-NST**K**NMK	τ91	τ91	503	139	32.74
ITG**K**EFQR-NPAI**K**IR	τ138	τ138	362	252	32.66
LSMVGFPLLY**K**YK-ANPFA**K**K	τ95	τ95	243	251	32.5
LS**K**LAEGDSVFEGPLMEER-ITA**K**VVDKYEK	τ131	τ131	702	627	32.38
**K**IIFLPTVGEDAIQR-YMGSTTTLD**K**K	τ138	τ138	619	592	32.27
LL**K**DLSSAR-LLLLA**K**VK	τ91	τ91	161	186	32.21
QV**K**NSTNASVAGNISNPK-TPN**K**NK	τ138	τ138	491	483	32.15
SGE**K**GINTMDLAQVTGQDPR-QLKL**K**K	τ138	τ138	123	170	31.74
IYDFEGK**K**K-ITG**K**EFQR	τ138	τ138	401	362	31.6
STSVNVSDG**K**IK-TTVVVENT**K**EDK	τ138	τ138	708	729	31.51
IYDFEG**K**KK-ITG**K**EFQR	τ138	τ138	400	362	30.59
AFT**K**SSEYYLESVDK-Q**K**ENTGGYR	τ138	τ138	370	383	30.51
NSTNASVAGNISNP**K**R-IPPNS**K**K	τ138	τ138	506	478	30.43
NSSATQ**K**SLR-L**K**KNSTK	τ91	τ91	598	134	30.22
IYGLN**K**EK-NTVQ**K**LR	τ91	τ91	178	130	29.81
IPPNS**K**K-TPN**K**NK	τ138	τ138	478	483	29.65
EASNF**K**VK-G**K**SYGR	τ131	τ131	102	113	27.62
GTYI**K**NYQLFVHDLSDK-ANPFA**K**K	τ95	τ95	266	251	27.42

* Cross-linked lysine residues are underlined and in bold

** Peptides are listed in descending order of ld Score, only cross-links with an ld score > 25 were selected for analysis

Supplementary Table 5: Data collection statistics

	τ131 SeMet $P6_2$	τ131 SeMet $P4_3$	τ131 Hg $P4_3$	τ138 s-SAD
**Data collection**				
Beamline	ESRF (ID23-1)	ESRF (ID29)	ESRF (ID14-4)	Home Source
Space group	$P6_2$	$P4_3$	$P4_3$	H32
Cell dimensions				
a=*b*, *c* (Å)	121.71 97.78	106.01 99.11	104.99 97.49	129.05 68.11
Wavelength (Å)	0.97924	0.9793	1.00650	1.5418
Resolution (Å)*	50-4.5 (4.61-4.50)	80-4.6 (4.85-4.6)	80-4.2 (4.43-4.2)	50-1.86 (1.91-1.86)
CC ½ (%)	0.99 (0.47)	0.99 (0.70)	0.99 (0.63)	0.99 (91.8)
R_{merge} (%)	9.6 (183.3)	14.3 (85.1)	10.8 (51.8)	5.9 (63.7)
I/σI	16.7 (1.6)	8.4 (2.3)	9.3 (2.0)	40.3 (4.6)
Completeness (%)	99.1 (89.9)	99.9 (100.0)	99.9 (100.0)	99.9 (99.6)
Redundancy	20.3 (16.6)	3.7 (3.8)	4.1 (1.8)	15.6 (13.6)
Sites	7 Se	8 Se	6 Hg	6 S

* Values in parentheses correspond to the highest-resolution shell
